# Molecular genetic analyses of the N, NSm and NSs genes of a local population of Orthotospovirus tomatomaculae reveal purifying selection in crops in the southeastern USA

**DOI:** 10.1099/jgv.0.002119

**Published:** 2025-07-07

**Authors:** Bhavya Shukla, J. Michael Moore, Theodore McAvoy, Nino Brown, Albert K. Culbreath, Sudeep Bag

**Affiliations:** 1 Department of Plant Pathology, University of Georgia, Tifton, GA, 31793, USA; 2 Department of Crop and Soil Sciences, University of Georgia, Tifton, GA, 31793, USA; 3 Department of Horticulture, University of Georgia, Tifton, GA, 31793, USA

**Keywords:** BEAST analysis, *Orthotospovirus tomatomaculae*, phylogenetic analysis, purifying selection

## Abstract

*Orthotospovirus tomatomaculae* [tomato spotted wilt virus (TSWV)] is a major pathogen in horticultural and row crops worldwide including the USA. In this study, tomato spotted wilt disease incidence was monitored in *Arachis hypogaea* (peanut; year 1990 to 2024) and *Nicotiana tabacum* (tobacco; year 2000 to 2024) in commercial farmers’ fields in the Southeastern USA. Furthermore, nucleocapsid (N), nonstructural movement (NSm) and nonstructural silencing suppressor (NSs) protein gene sequences of TSWV global populations from North America, South America, Europe, Asia-Pacific, Africa and Australia were compared with local US population and analysed to understand the genetic variability in the virus genome. In our study, full-length sequences of 94 N, 111 NSm and 78 NSs genes were amplified from TSWV-infected *A. hypogaea* (peanut), *Capsicum annuum* (pepper), *N. tabacum* (tobacco) and *Solanum lycopersicum* (tomato). nt-based phylogenetic analysis of N, NSm and NSs genes correlated with the geographical location of the TSWV isolates, with notably higher substitution rates in the population of recent years. In addition, the least genetic variability was observed in the N gene of the local population upon comparison with other global TSWV population. The neutrality test of TSWV suggested a non-neutral evolution of the virus genome. Low variation among the selected genes might be attributed to strong purifying selection pressure in the populations. Furthermore, estimation of selection pressure (dN/dS) on small (S) segment-encoded N protein and nonstructural protein showed higher purifying selection than the movement protein encoded by the medium (M) segment of the TSWV isolates. Single-likelihood ancestor counting suggested an overall negative selection pressure on several codons of the selected genes, which indicated that natural selection and population bottleneck events might have influenced the evolution of TSWV. Our study also deciphered high gene flow and low genetic differentiation amid the different TSWV population sets. Additionally, BEAST analysis of TSWV N gene sequences from GA predicted the most common recent ancestor existed ~25 years ago. This data was further correlated with disease incidence data from peanut and tobacco crops obtained in the last three decades. These findings suggest the intermixing of TSWV isolates between peanut, pepper, tobacco and tomato crops, while the virus genome has undergone strong purifying selection.

## Data Availability

All the sequences generated were submitted to the NCBI GenBank and will be available freely on the publication of the manuscript. The accession numbers were included in the manuscript and Table S2.

## Introduction

*Orthotospovirus tomatomaculae* [formerly known as tomato spotted wilt virus (TSWV)] is a major threat to horticultural and row crop production including peanut (*Arachis hypogaea*), pepper (*Capsicum annuum*), tobacco (*Nicotiana tabacum*) and tomato (*Solanum lycopersicum* L.) in the southeastern USA. With an elaborative host range of more than 1,000 plant species, it poses a grave danger to global food security. The reasons attributed to the flourishment of TSWV are the favourable environment for its vector (thrips species), cultivation of its major hosts worldwide [1] along with the ability to rapidly adapt with the help of high genetic diversity and positive pleiotropy [2]. TSWV is a representative member of the genus *Orthotospovirus*, family *Tospoviridae* comprising 28 virus species (https://ictv.global/, 2025 Release, MSL #40). Its genome is ambisense, composed of three L, M and S RNA segments (large, 8.9 kb; medium, 4.8 kb; and small, 2.9 kb). These segments encode a total of five proteins, namely, RNA-dependent RNA polymerase (L segment), nucleocapsid (N) protein (S segment), glycoproteins (M segment) in negative sense and two other nonstructural proteins (M and S segments) in positive sense [3]. One of the nonstructural proteins encoded by nonstructural movement (NSm) gene was reported to be involved in cell-to-cell movement, symptom development [4] and effector gene for Sw-5b (R) gene in tomato [5]. The regions involved in tubule formation, cell-to-cell movement and foliar necrosis are the aa residues in the amino terminal (N-terminal) of the protein [6]. In addition, the nonstructural silencing suppressor (NSs) gene product is involved in suppressing the host’s RNA interference mechanism. The role of the N-terminal domain is proven to be essential for silencing suppressor and avirulence activity, whereas the carboxy terminal (C-terminal) domain has structural significance [7]. TSWV is transmitted in a propagative and circulative manner by nine different species of thrips [8], with *Frankliniella occidentalis* and *Frankliniella fusca* majorly responsible for TSWD epidemics in GA (https://tswv.caes.uga.edu/usda-ramp-project/thrips-vectors.html). Vector has shown to be a major bottleneck in the evolution of a virus [9]. Contrastingly, thrips species (specifically *F. occidentalis*) was observed to actively increase the diversity of TSWV. This could be attributed to the ability of TSWV to replicate inside its thrips vector [2].

TSWV is a causal agent of tomato spotted wilt disease (TSWD). The symptoms of TSWD are diverse and vary with its hosts. Common symptoms include stunting of plants in early infection, while chlorotic or necrotic rings on the foliage and fruit in late infection. Therefore, both the quality and quantity of agricultural produce are severely affected [10]. In peanut, an extensive integrated pest management tool, Peanut-R_x_ (https://peanuts.caes.uga.edu/extension/peanut-rx.html), was developed for growers to reduce the impact of TSWD epidemics on peanut yields. An important tool in the risk index is the usage of cultivars with robust defence-related response upon TSWV infection [11]. Due to the absence of resistant cultivars with good agronomic traits in tobacco, TSWD is managed by controlling thrips with the use of imidacloprid and acibenzolar-S-methyl for boosting plant immunity in southeastern GA, USA (https://tswv.caes.uga.edu/tobacco.html). Cultivation of resistant varieties is the primary management strategy utilized in crops with single-gene resistance against TSWV, such as *Sw-5b* in tomato [12] and *Tsw* in pepper [13]. Even with these different approaches to control TSWD, it is still a significant issue for sustainable production of these crops in the Southeast. In addition, the global emergence of resistance breaking (RB) isolates [14,16], via local evolution [17], was reported in both tomato and pepper crops. Specific point mutations in NSm (effector gene in tomato) and NSs (effector gene in pepper) genes are associated with RB isolates of TSWV [18,19], capable of causing a global pandemic.

The presence of row and horticultural crops with different management strategies within the same growing season in a region provides an excellent environment for TSWV to adapt. The RNA nature of the TSWV genome plays an important role in increasing its evolutionary fitness [20]. Therefore, a vigilant approach to prevent the emergence of virulent strains of TSWV is important for the sustainability of management strategies. Earlier studies on TSWV phylogeny and population studies have been conducted on the aforementioned crops [21,23]. However, studies contrasting the molecular diversity between TSWV isolates from peanut, pepper, tobacco and tomato crops are lacking. Furthermore, NSm and NSs genes of TSWV-RB isolates were studied to identify their origin [24,25]. Therefore, we conducted this study to analyse the diversity of N, considered to be the most conserved among all the five genes of TSWV, NSm and NSs genes. The NSm and NSs genes were selected because of their role as avirulent genes in tomato [5] and pepper [26], respectively, for resistance against TSWV. In addition, they probably undergo specific mutations to adapt to different hosts [2]. Thus, the investigation of nt diversity present in these genes with different sets of adaptive pressure such as monoculture of resistant/moderately resistant cultivars would provide insights of TSWV adaptation. Our study incorporated symptomatic TSWV samples from the years 1997, 2018–20 and 2022–24 to explore the diversity in genes/ORFs. Furthermore, full-length N, NSm and NSs gene sequences were retrieved from the NCBI GenBank public database. Accordingly, analyses were performed to understand the host impact on TSWV strain emergence.

## Methods

### Disease incidence monitoring of peanut and tobacco

TSWD incidence and yield loss data of peanut were collected for the last three decades (year 1992 to 2024) from commercially grown peanut crops following standardized survey procedures in two to five fields in each county (five to ten counties each year) performed by the University of Georgia (UGA) Agriculture and Natural Resources (ANR) Agents. This study was performed by the UGA, Peanut Research Team (https://peanuts.caes.uga.edu/team-members.html), and yield loss was estimated [27,28]. Similarly, disease incidence (DI) data was collected by the UGA Tobacco Research Team (https://tobacco.caes.uga.edu) for more than two decades (year 2000 to 2024) from commercial tobacco crop fields located in GA, USA [29].

### Plant sample collection

Symptomatic peanut, pepper, tobacco and tomato samples with TSWD-like symptoms were collected in the summer and fall growing seasons. Samples collected from the years 1997, 2018, 2019, 2020 and 2022 were cleaned with distilled water, dried at room temperature and lyophilized. Further, the leaf tissues from 1997 and 2018–22 were stored in 4 and 25 °C until processing. All the symptomatic leaf samples (collected in 2023–24) were acquired from growers’ farms located in different counties of southeast GA, USA. The leaf samples were brought in ice coolers to the UGA Crop Virology Laboratory on the Tifton Campus. Further, they were surface cleaned and stored in −80 °C until processing for RNA isolation.

### Total RNA extraction and cDNA synthesis for TSWV genes

Total RNA was extracted from 30 mg of symptomatic leaf tissues using the Quick-RNA Miniprep kit (Zymo, Irvine, CA, USA) following the manufacturer’s instruction with minor modifications. Infected tissues were ground manually by adding liquid nitrogen followed by the addition of 600 µl of lysis buffer. Each sample was processed individually and purified using the column provided in the Quick-RNA Miniprep kit. Samples were eluted in 30 µl of RNase-free water. The RNA samples were further checked for integrity and quantity using a NanoDrop spectrophotometer (Thermo Fisher Scientific, Waltham, MA, USA). Only samples with OD_280/260_ ranging from 1.8 to 2.0 and OD_260/230_ ranging from 2.0 to 2.2 were processed for cDNA preparation. For cDNA preparations, 2 µg of total RNA was used as an initial template followed by adding individually 10 µM of N, NSs and NSm gene-specific reverse primers (Table S1, available in the online Supplementary Material) and 200 U of SuperScript IV reverse transcriptase (Invitrogen, Carlsbad, CA, USA). The cDNA was used as a template for PCR reactions to amplify N, NSm and NSs genes of TSWV.

### PCR amplification of full-length TSWV genes

To amplify the full length of N and NSm genes, one pair of primers was utilized, whereas for NSs gene, three pairs of primers were used (Table S1). PCR assay was performed by adding 20 µl of PCR reaction mix comprised of 10X reaction buffer, 2 µl (~200 ng) cDNA template, 5X dNTPs, 1X MgSO_4_, 10 pmole µl^−1^ of each forward and reverse primers, 1U of Taq high-fidelity DNA polymerase (Thermo Fisher Scientific) and deionized water. PCR cycling conditions were set as pre-denaturation at 95 °C for 3 min, followed by 32 PCR cycles with denaturation at 95 °C for 30 s, annealing for 45 s, extension at 72 °C for 1 min and final extension at 72 °C for 7 min. The PCR products were analysed by visualizing in 1% agarose gel and compared with the expected size of the amplicons of the 1 kb plus DNA ladder (Thermo Fisher Scientific) (Fig. S1, Supplementary Material 1).

### Sanger’s sequencing of amplified TSWV genomic components

Amplicons of N (909 bp), NSm (1,162 bp) and NSs (2,369 bp) genes (comprising three overlapping fragments: fragment-I of 768 bp, fragment-II of 700 bp and fragment-III of 901 bp in length) were eluted out from agarose gel using QIAquick PCR purification Kit (Qiagen, Germantown, MD, USA) according to the manufacturer’s instructions. Purified PCR products with 10–30 ng µl^−1^ of DNA concentration were sent for bidirectional Sanger’s sequencing at Eurofins Genomics (Eurofins MWG Operon Inc., Louisville, KY, USA) sequencing facility. The obtained full-length sequences of N, NSm and NSs genes were manually checked and aligned using the BioEdit (v7.0.5.3) [30] sequence alignment editor tool. Furthermore, ORFs were checked using the NCBI ORF finder (https://www.ncbi.nlm.nih.gov/orffinder/). The obtained 283 sequences were submitted to the NCBI GenBank (Table S2) and hereafter were referred to as the TSWV-Georgia (TSWV-GA) isolates. Pairwise nt identity and recombination in all the sequences utilized in this study were assessed using Sequence Demarcation Tool (SDT) v1.3 [31] and Recombination Detection Program (RDP) v5 [32].

### Understanding the evolutionary dynamics of TSWV

Full-length sequences of N (*n*=47), NSm (*n*=59) and NSs (*n*=56) genes of TSWV were retrieved individually from the NCBI GenBank as available on September 2024 (Table S2) and were aligned with sequences of the TSWV-GA isolates generated in this study using the multiple sequence alignment with muscle algorithm in mega-X (v10.2.4) tool. A phylogenetic tree of N, NSs and NSm gene sequences was constructed using the maximum-likelihood (ML) statistical method with 1,000 replicates of bootstrap, including Tamura–Nei model for substitution with uniform rates, and nearest neighbour interchange as ML heuristic method [33] with mega-X software. For simplicity, the phylogenetic tree was visualized using the Interactive Tree of Life (iTOL) v6.0 tool [34].

### Estimation of genetic diversity, selection pressure, gene flow and genetic differentiation

The genetic diversity of N (*n*=118), NSm (*n*=187) and NSs (*n*=162) genes of TSWV was analysed (Table S2) using DNA Sequence Polymorphism (DnaSP6) v6.12.03×64 tool [35]. The analysis was performed by generating the number of segregating sites (S), haplotype number (h), haplotype diversity (Hd), average number of nt differences (k), average nt diversity (π), total number of mutations (η; Eta) and Watterson’s theta (θ-w) coefficient. Broadly, all the population sets (eight total) were categorized into two groups, namely, global and local. The global group contained four population sets with all the representative sequences from the USA and the world, along with GA isolates for peanut, pepper, tobacco and tomato, whereas the local group contained the same four sets, for each crop with the sequences of only GA isolates (TSWV-GA). The neutrality tests were performed with Tajima’s D test, Fu and Li’s D and F statistics. These tests were performed for both local and global populations of TSWV. The population differentiation was measured with the help of statistical tests including Ks*, Kst*, Z* and Snn factors. Gene flow was measured using Fst and Nm statistics with a permutation test with 1,000 replications. Statistical analysis was analysed as *, 0.01<*P*<0.05; **, 0.001<*P*<0.01; and ***, *P*<0.001, and ns represents non-significant.

### Estimation of non-synonymous and synonymous substitution-based selection pressure on codons of different protein coding genes (N, NSm and NSs) of TSWV

All the nt sequences (Table S2) of N, NSm and NSs genes obtained from each crop (peanut, pepper, tobacco and tomato) were analysed for estimating selection pressure using single-likelihood ancestor counting (SLAC) method using Data monkey (https://www.datamonkey.org) online platform [36]. Each gene was analysed individually, and the dataset for each crop contained TSWV isolates from the US and TSWV-GA isolates. aa sequences of N, NSm and NSs genes of TSWV-GA isolates were aligned for mutation analysis using clustalw in BioEdit.

### Recent ancestral analysis of TSWV using *in silico* based cross-platform programme

A phylogeny-based Bayesian analysis was performed using Bayesian Evolutionary Analysis Sampling Trees (BEAST X v10.X) software package [37] employing Markov chain Monte Carlo (MCMC) algorithm, allowing for robust evolutionary inferences. The sequences were analysed for temporal signal using IQ-TREEv1.6.12 [38] and TEMPoral Exploration of Sequences and Trees (TempEST) software [39]. The generalized time reversible model was chosen for the analysis as it allows for different rates of substitution between all the pairs of nt of the genome. Additionally, a gamma site heterogeneity model was selected to encompass substantial rate variation across the sites. The genome sequences and nt substitution model parameters were exported in BEAUTi2: Standard [40]. Each gene dataset contained sequences of TSWV-GA isolates in addition to representative TSWV-US (Table S2). The taxa were generated based on crop (peanut, pepper, tobacco and tomato), and N, NSs and NSm genes were analysed separately. A strict clock model was selected, and the coalescent constant population model was set as the tree prior under the default assumption of uniform distribution of taxon sets. The MCMC was set to run at 75,000,000 with a trace log generated for every 1,000 trees to achieve an effective sample size of >200. Tracer v1.7.1 [41] was used to visualize the log files generated by the BEAST software.

## Result and discussion

### Variable DI in peanut and tobacco crops over the decades

**Fig. 1.**
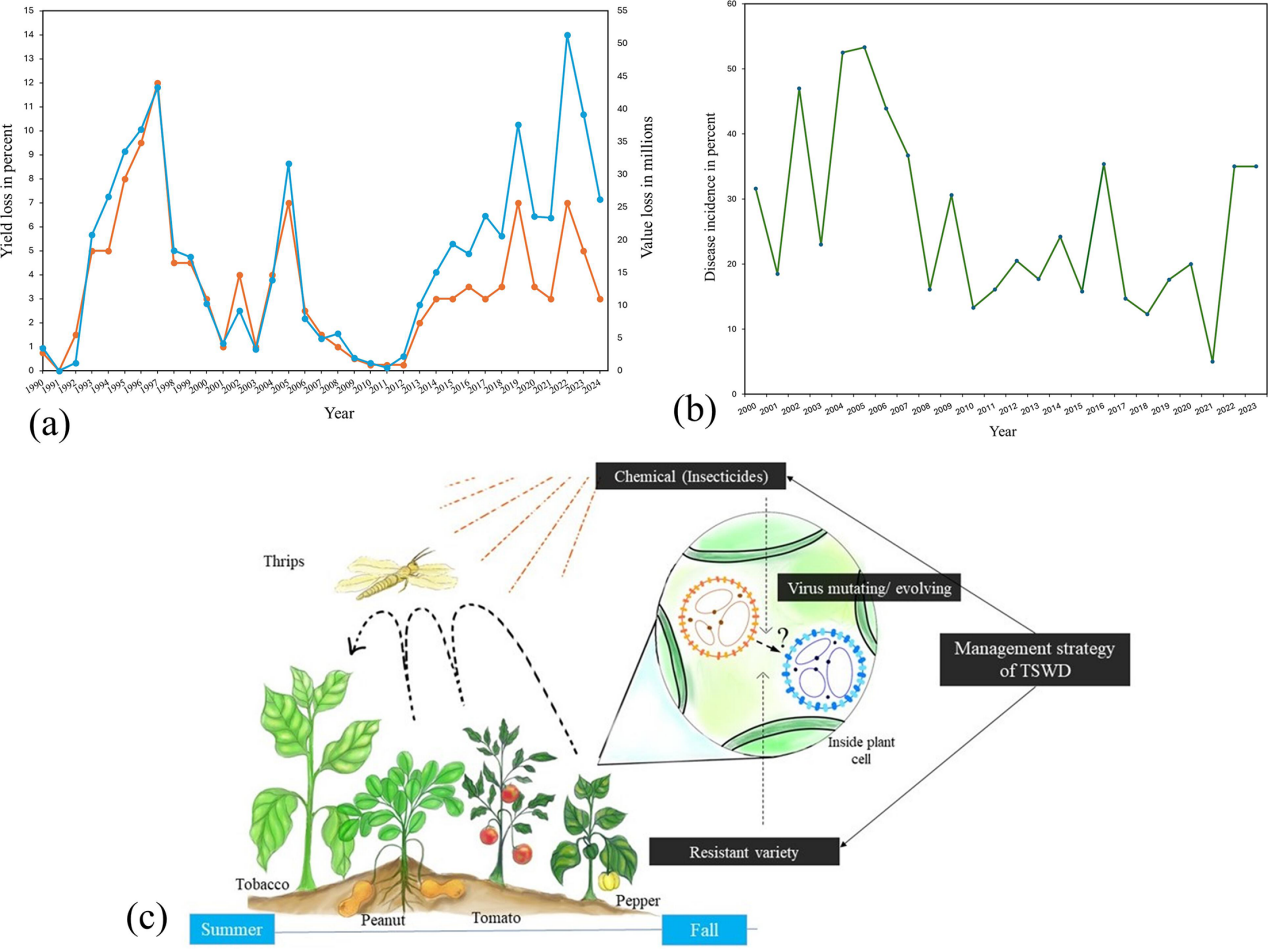
(a) Obtained graph representing per cent yield loss in commercially grown peanut in the range of 0–15% (in y-axis, left side panel) and value loss in million dollars (in y-axis, right side panel) in comparison with time represented in years (x-axis). The data here represents the total loss due to TSWD from 1990 to 2024. (b) Graph shows per cent DI in commercially grown tobacco in GA, USA. The y-axis represents the per cent DI, whereas the x-axis shows the year of occurrence from 2000 to 2023. (c) Schematic representation of the proposed hypothesis for this study. The hypothesis suggests that management strategies such as cultivation of both horticultural host crops (with single gene resistance) with row crops (with no single gene resistance) in the same environment with excessive implication of insecticides on the vector (thrips) are playing a role in selecting the mutations already present in TSWV population in Southeastern GA, USA.

The DI rating was derived from the consistent monitoring of symptoms observed since the pathogen was introduced in the region, and the yield loss data in peanut was based on TSWD incidence reported in the last three decades. Yield loss in GA has been variable across the decades in peanut, with the highest peaks in the years 1997 (12% yield loss; ~$45 million value loss) and 2005 (~7% yield loss; ~$33 million value loss). More recently, in 2019 and 2022, severe losses have again been evident, with ~$24 million lost in both years to TSWD. With an increase in DI, moderately resistant peanut cultivars such as ‘Georgia Green’ [42] and ‘Georgia-06G’ [43] were introduced in 1997 and 2006, respectively. Along with the cultivation of moderately resistant cultivars, growers also adopted cultivation practices as recommended in Peanut R_x_ [44]. A similar trend was visible in DI data for tobacco with a peak in 1999 (~40% incidence; 8% yield loss), 2002 (47% incidence; 20% yield loss) and 2005 (53.3% incidence; 18% yield loss). Since 2005, the DI remained constantly above 12% (except in 2021), peaking at 35% in 2023–24. With no resistant varieties in tobacco, growers heavily depended on insecticide (Admire Pro; imidacloprid) and plant immunity boosters (Actigard; acibenzolar-S-methyl) usage in the greenhouse, herbicide application at least 14 days before pre-transplant, transplanting after 7th of April and growing less susceptible varieties of tobacco (NC 196) [45]. Even with an extensive integrated management strategy, TSWD is still a persistent and economically significant problem in the Southeast (Fig. 1a, b).

An amalgam of various factors collectively influences disease prevalence in a region including pathogen as a pivotal factor. As a result, we correlated the recent increase in DI (especially 2013 onwards) with the emergence of TSWV-RB strains [17,24]. Therefore, we hypothesized that the variable DI in row crops with no or moderate resistance could be due to overlapping cultivation periods of crops bearing TSWV resistance such as pepper and tomato. The probability of intermixing of TSWV isolates from pepper/tomato (carrying *Sw-5b*/*Tsw* resistance gene) to tobacco (absence of any known resistance gene) and peanut (with quantitatively inherited resistance) [46] crops increased due to high density of its vector’s (western flower thrips, *F. occidentalis*, and tobacco thrips, *F. fusca*) population in the field [23]. Such a scenario might lead to the evolution and emergence of novel variants in the southeastern US crop production rotation (Fig. 1c). Hence, we chose to investigate the diversity and population dynamics of TSWV isolates from these selected crops.

### Sample collections from four major hosts exhibiting diverse TSWD symptoms

**Fig. 2.**
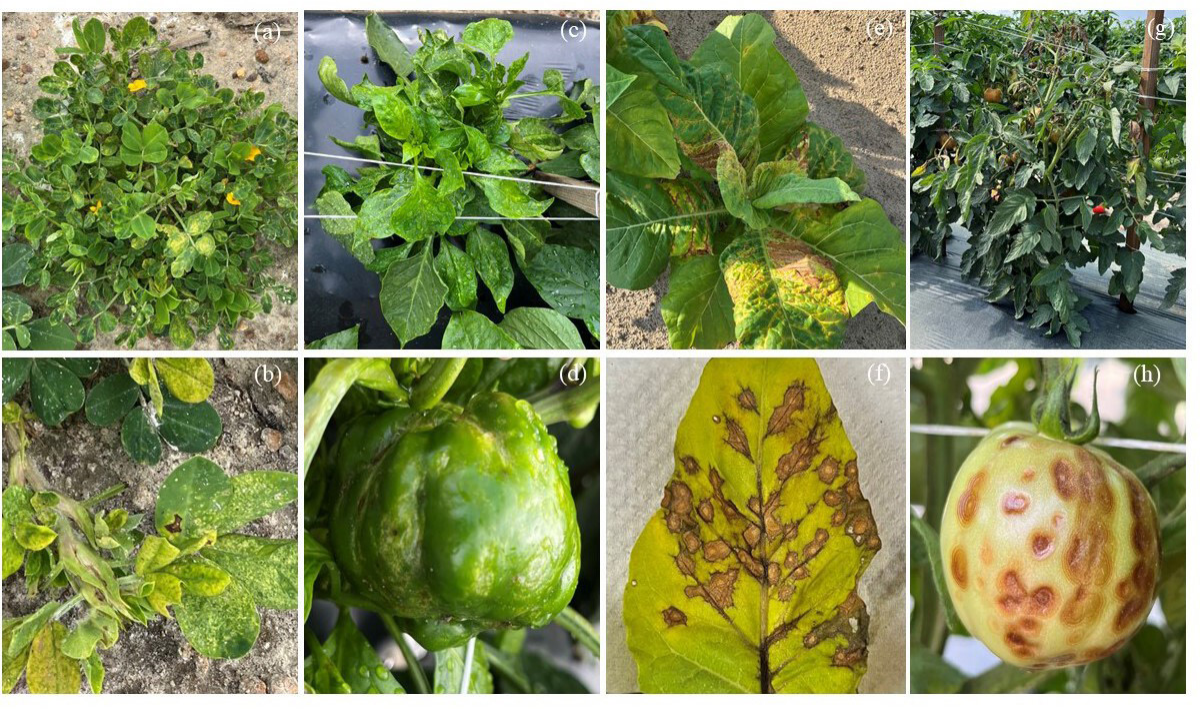
Symptoms of TSWD in commercially grown row and horticultural crops in natural conditions. (**a, b**) Peanut plants with chlorotic ringspots on the lower leaf foliage and curled disfigured on the uppermost leaves and reduced height (stunting). (**c, d**) Symptomatic pepper plants were showing puckered leaves, stunted growth and characteristic chlorotic ringspots on fruits. (**e, f**) Tobacco plants were showing chlorosis, necrotic and chlorotic ringspots on the leaves, large necrotic patches and necrotic lesions on the stem. (**g, h**) Tomato plants with stunted growth, wilting symptoms, leaves with bronzed appearance, small brown spots or ringspots on the leaves and concentric necrotic ringspots on the fruits.

Symptomatic samples from peanut (*n*=54), tobacco (*n*=52), tomato (*n*=41) and pepper (*n*=19) were collected from 1997 and 2018 to 2024 (Table 1). The samples were collected in the spring and summer season for pepper, tobacco and tomato, whereas peanut samples were collected during the fall season. Stunted peanut plants with chlorotic ringspots on the lower leaf foliage and curled, disfigured leaflets from the uppermost leaves (Fig. 2a, b) were collected. In pepper plants, puckered leaves, stunted growth and characteristic chlorotic ringspots (Fig. 2c, d) on the leaves were acquired. Tobacco plants with chlorosis, necrotic, chlorotic ringspots on the leaves, large necrotic patches and necrotic lesions on the stem (Fig. 2e, f) were collected from research plots at the UGA Tifton Campus and farmer’s fields. Similarly, symptomatic tomato plants with stunted growth, wilting symptoms, leaves with bronzed appearance, small brown spots or ringspots on the leaves and concentric necrotic ringspots on the fruits (Fig. 2g, h) were collected. The presence of disease in horticultural and row crops and in susceptible or moderately resistant varieties was widespread.

**Table 1.** Tabular representation of time and location of sample (symptomatic leaf tissues of peanut, pepper, tobacco and tomato plants) collection, along with the number of sequences (N, NSm and NSs genes of TSWV) obtained

Crop	Year of collection	County (state)	Total no.of symptomaticsamplesprocessed	Sample ID		
				**N**	**NSm**	**NSs**
**Peanut**	1997	Columbia, Fayette, Jeff Davis, Laurens, Pierce, Tift and Worth (GA)	13	1 to 11, 13	1–6, 10–13	1–3, 5, 8, 10–12
	2020		10	2, 3, 5, 6, 7, 9, 10	1, 3, 4–6	1–7, 10
	2022		6	1 to 6	0	1 to 6
	2023		10	1 to 8, 10	1 to 10	1, 2, 4, 5, 6–8, 10
	2024		15	1, 2, 4, 6–8, 11–13, 15	1, 2, 4, 6–8, 11–13, 15	1,2,4,6–8,11,12
**Total**			**54**	**44**	**35**	**38**
**Tobacco**	2018	Atkinson, Berrien, Candler, Colquitt, Evans and Tift (GA); Alachua, Lafayette and Suwannee (FL)	10	0	1 to 5	0
	2019		10	4, 5	1 to 7	0
	2020		10	5, 6	1 to 4	0
	2023		10	1 to 10	1, 2, 4–10	1, 2, 4, 7–10
	2024		12	1–3, 5–9, 11, 12	1–3, 5–9, 11, 12	1–3, 5–9, 11, 12
**Total**			**52**	**24**	**35**	**17**
**Tomato**	2019	Colquitt, Grady and Tift (GA)	10	0	1	0
	2020		5	0	2	0
	2023		16	1–3, 5–8, 12, 13	1–8, 10–17	1 to 9
	2024		10	1 to 10	1 to 10	1 to 10
**Total**			**41**	**19**	**29**	**19**
**Pepper**	2019	Colquitt and Tift (GA)	10	0	1, 2	0
	2020		5	2, 3, 4	1 to 6	0
	2023		4	1, 2, 3, 4	1, 2, 3, 4	1, 2, 3, 4
**Total**			**19**	**7**	**12**	**4**
**Total**			**166**	**94**	**111**	**78**

### Amplification of full-length N, NSm and NSs sequences from infected samples

A total of 166 symptomatic samples comprising peanut, pepper, tobacco and tomato crops were used for virus detection using PCR and TSWV Immunostrips. Forty-four N, 35 NSm and 38 NSs genes of TSWV from peanut (years 1997, 2020 and 2022–24); 24 N, 32 NSm and 17 NSs genes of TSWV from tobacco (years 2018–20 and 2023–24); 19 N, 29 NSm and 19 NSs genes from tomato (years 2019–20 and 2023–24); and 7 N, 12 NSm and 4 NSs genes from pepper (years 2019–20 and 2023) were amplified and sequenced (Table 1).

### Phylogenetic analysis reveals significant spatial influence

**Fig. 3.**
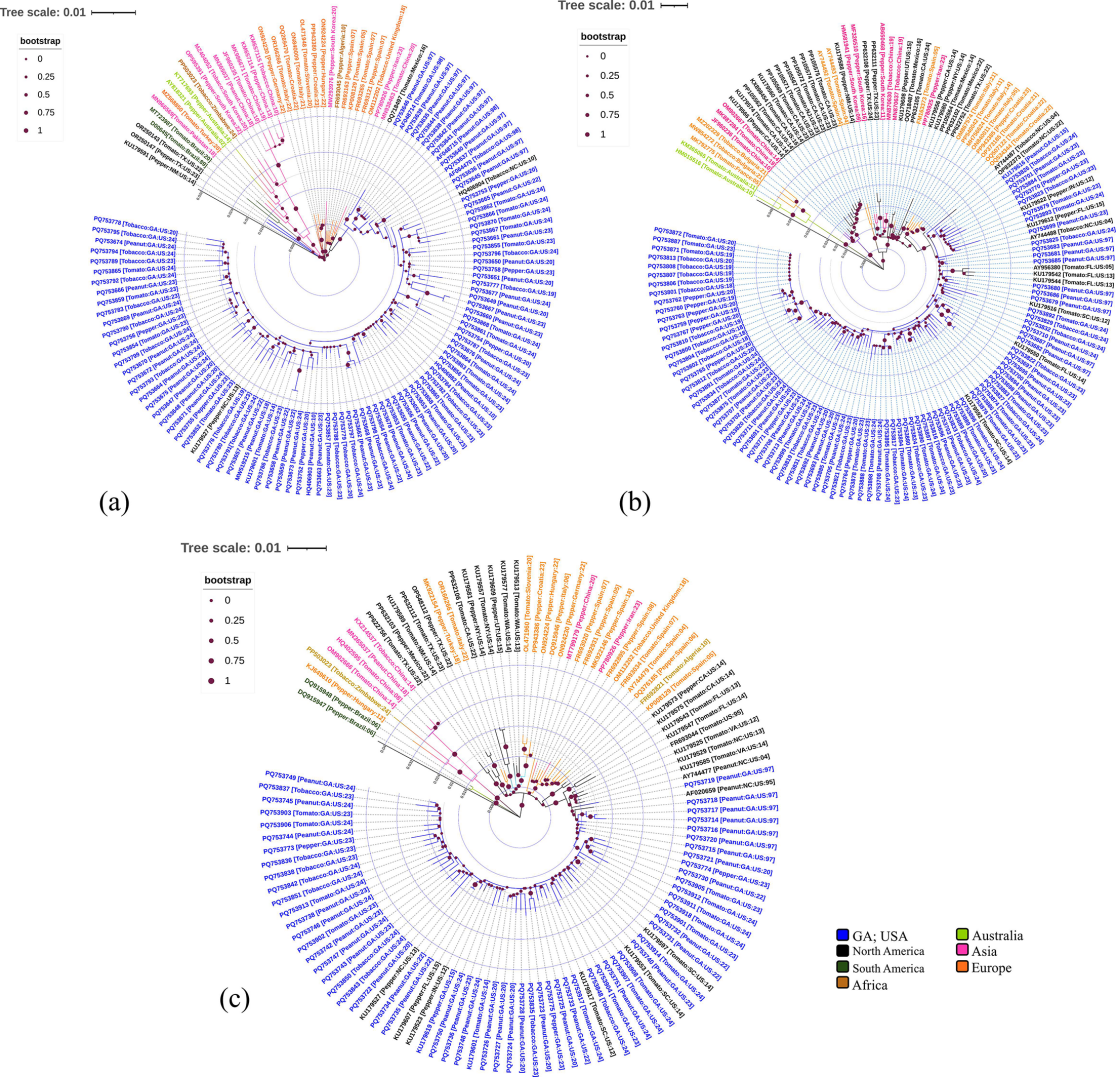
(a) Phylogenetic dendrogram of TSWV encoding N gene. Fifty-six full-length N gene nt sequences from peanut, pepper, tobacco and tomato crops were retrieved from the NCBI GenBank. Sequences were aligned by muscle algorithm, and a phylogenetic tree of the N gene was generated with the ML-based method incorporating the Tamura–Nei model with the bootstrap of 1,000 replicates using the mega-X tool. Colour coding indicates the geographic location of TSWV isolates, whereas the distance scale bar indicates the rate of substitution per site per year. (b) Fifty-nine full-length NSm gene nt sequences from peanut, pepper, tobacco and tomato crops were retrieved from the NCBI GenBank. Sequences were aligned, and a phylogenetic tree of NSm was generated similarly as used for N gene using mega-X tool. Colour coding and distance scale bar were indicated as mentioned earlier. (c) Fifty-six full-length NSs gene nt sequences from peanut, pepper, tobacco and tomato crops were retrieved from the NCBI GenBank. Sequences were analysed in a similar fashion as mentioned earlier. For a better visualization of the trees, images were depicted using iTOL v6.0 tool [34].

Phylogenetic analysis of TSWV N gene with 94%–100% pairwise nt identity (Fig. S2a) showed the presence of at least five major clades (Fig. 3). Broadly, Clade I comprised sequences from the USA (TX and NM), whereas Clade II contained sequences from southern and eastern Asia, Australia, South America and southern Africa, suggesting global emergence of TSWV around the world. Indeed, Clade II was further classified into four minor sub-clades (Clade II a–d) based on geographical dispersal of TSWV. Clade III consists of seven sequences and confirmed the presence of TSWV in Croatia, Germany, Hungary, Italy and Slovenia. This clade consists of N gene sequences from TSWV isolates originated only in Europe. In Clade IV, nine representative sequences were aligned with sequences obtained from African, Chinese, Iranian, South Korean, Spanish and UK isolates. Like Clade III, Clade V has 102 sequences, and all were majorly reported from the USA (GA and NC). All the TSWV-GA isolates were encompassed within Clade V, suggesting a monophyletic origin of N genes in GA. In addition, peanut TSWV-GA isolate (PQ753635–PQ753642 and PQ753644–PQ753646; peanut; 1997) showed close relatedness with peanut, tobacco and tomato isolates reported in the year 1998 in GA. Therefore, N gene-based phylogenetic relatedness might be correlated with the geographical distribution of TSWV isolates in these cultivated crops. Phylogenetic distance scale (>0.016 nt substitution/site/year) of the recent TSWV-GA isolates from all four crops suggested a higher nt substitution rate in the last two decades upon comparison with the peanut TSWV-GA isolate of 1997.

Phylogenetic dendrogram of TSWV-NSm gene sequences with 91%–100% pairwise nt identity (Fig. S2b) had three major clades with a significant difference in the number of sequences representing these clades (Fig. 3b). Clade I consisted of sequences from Australia and Europe, whereas Clade II contained the majority of isolates from the USA (CA and NJ) and China. However, the third clade had sequences from Europe, Asia and North America. TSWV-GA isolates were closely related to other isolates of TSWV identified in Italy and Croatia. The TSWV-US isolates reported from the southeastern region including SC, NC, FL and GA were depicted in proximity and therefore present in the same clade. N gene sequences of TSWV-GA isolates were least related to TSWV-Brazilian isolates, whereas closely related to the TSWV isolates reported from Europe. Unlike the N gene sequences of TSWV isolates from 1997, NSm gene sequences did not form a separate branch and share clades with relatively more recent isolates (Fig. 3b). The highest phylogenetic distance (>0.048 nt substitution/site/year) of NSm suggested a maximum variation in tomato infecting TSWV-AU from Australia in an RB isolate as compared with other TSWV isolates. The RB strains from CA (PP105564, PP105569, PP105571, PP105572, PP105574 and PP105575) were grouped with TSWV WT isolates from China (JF960236, 2010; OM902667, 2014; and MK887284, 2019) and CA (KU179560, KU179566 and KU179574, 2014 and KX898454, 2016) in Clade II. Grouping of RB strains with WT isolates demonstrated its local emergence. Further, the presence of RB strains in two different clades demonstrated its polyphyletic origin. The phylogenetic distance of NSm sequences from RB strains from CA was 0.024> and >0.018 nt substitution/site/year. This demonstrated higher geographical influence than the R gene in tomato cultivars.

The ML phylogenetic tree of NSs gene sequences with 93%–100% pairwise nt identity (Fig. S2b) indicated the presence of three major clades. Clade I consisted of TSWV sequences from Brazil, whereas Clade II contained sequences from China, Hungary and Zimbabwe, and Clade III contained sequences from Europe, the USA and Asia (Fig. 3c). TSWV-GA shared phylogenetic relatedness with other isolates previously reported from GA and other states of the USA (FL, IN, NC and SC). The highest phylogenetic distance (>0.04 nt substitution/site/year) suggested maximum variation in TSWV tobacco isolate from Zimbabwe as compared with other TSWV isolates utilized in this study. Our study showed that spatial influence was evident in phylogenetic analysis, which has also been observed by others [21,25, 47, 48]. TSWV isolates from CA were grouped together with Chinese isolates based on NSm gene sequences. Similar observations were also reported by Macedo *et al*. [17].

TSWV isolates from North America including GA shared a high degree of genetic similarity with the isolates found in Europe, also reported by Tentchev *et al.* [47]. This suggests a common origin or exchange of genetic material over time, indicating possible movement or similar evolutionary path at both locations. N, NSm and NSs gene sequences from TSWV-TX and TSWV-NM isolates were present in proximity with TSWV-European isolates. Further, based on NSm gene, the lack of sequences of S segments from the same samples makes it challenging to interpret any reassortment event. Therefore, we infered that there is difference in the ancestral population of TSWV-CA and TSWV isolates from Southeast. Such observations also imply trans-continental dispersal of TSWV, also reported in other studies [47]. The monophyletic grouping of TSWV-GA isolates highlights their divergent origin and emphasizes their unique pattern of genome variability, reflecting distinct evolutionary behaviours. Such patterns were also observed with TSWV-Chinese isolates [21]. Unlike N gene, NSm and NSs genes displayed intermixing of population and absence of clade with just TSWV sequences from 1997. This indicated the presence of higher genetic diversity in NSm and NSs genes than N gene.

Furthermore, the influence of host (peanut, pepper, tobacco and tomato) on the phylogenetic relatedness of TSWV-GA isolates was not observed. Similar results were also established with TSWV-South Korean isolates [49] and TSWV-Turkish isolates [22]. The absence of clade differentiation based on crops suggested an intensive interchange of inoculum and insect vector between the crops. It could be attributed to the vector and overlapping growing seasons of the selected crops. This supported the proposed hypothesis of the transfer of TSWV isolates between the crops. But further analysis is needed to determine the cause of variable DI in different crops.

### Variation in nt diversity among TSWV genes across selected crops

Different population sets of TSWV isolates attained from peanut, pepper, tobacco and tomato crops were grouped to assess the diversity in TSWV-GA isolates. The pairwise nt diversity measuring an average number of nt differences per site between any two randomly chosen sequences in a TSWV population was represented with the π (Pi) value. The results revealed that the overall π values of global population sets were approximately three times higher than local population sets (Table 2a–c) of TSWV. This aligned with phylogenetic data depicting highly diverse sequences from the same crop of different regions. The N gene of TSWV-GA isolates was least diverse (Table 2a). In contrast, NSm gene sequences of TSWV-GA isolates showed the highest diversity (Table 2b), whereas the diversity of NSs gene sequences in the same population sets was intermediate between N and NSm genes (Table 2c). Globally, TSWV isolates from peanuts indicated the least genetic diversity (N, π=0.01219; NSm, π=0.01406; and NSs, π=0.0124), suggesting lesser variation in virus genomic components. This can be attributed to the higher purifying selection pressure on TSWV peanut isolates or the lack of TSWV sequences from countries other than China and the USA.

**Table 2.** Genetic diversity analysis of TSWV N, NSm and NSs genes

TSWV gene	Parameters used	Global population (*n*=178)				Local population (*n*=94)			
		Pepper	Tomato	Tobacco	Peanut	Pepper	Tomato	Tobacco	Peanut
**(a) N**	N	22	42	48	66	7	19	24	44
	h	22	41	45	56	7	18	24	36
	S	100	128	125	105	19	37	55	62
	η (Eta)	106	135	127	106	19	37	55	63
	π	0.02503	0.02305	0.02726*	**0.01219†**	0.00883	**0.00856†**	0.00941	0.00998*
	k	19.446	17.912	21.184	9.471	6.857	6.655	7.312	7.752
	θ-w	0.03826	0.04227	0.0399	0.03072	0.01016	0.01403	0.01979	0.01922
	Hd	1	0.999	0.996	0.993	1	0.994	1	0.99

		Global population (*n*=187)				Local population (*n*=111)			
**(b) NSm**	N	30	65	54	38	12	29	35	35
	h	30	61	49	36	12	26	31	33
	S	162	220	197	116	52	92	106	95
	η (Eta)	174	240	222	0.03378	53	96	117	104
	π	0.03168	0.03161	0.03238*	**0.01406†**	0.01505	0.01533	0.01742*	**0.01256†**
	k	28.8	28.738	29.432	12.784	13.682	13.938	15.837	11.413
	θ-w	0.05023	0.05902	0.0542	0.03278	0.01967	0.0274	0.03037	0.02701
	Hd	1	0.998	0.992	0.997	1	0.99	0.983	0.997

		Global population (*n*=161)				Local population (*n*=78)			
**(c) NSs**	N	29	55	35	42	4	19	17	38
	h	29	55	35	42	4	19	17	36
	S	202	324	279	226	37	104	115	175
	η (Eta)	211	348	297	240	37	107	116	182
	π	0.02062	0.02754	0.03703*	**0.0124†**	0.0135*	0.01084	0.01262	**0.0105†**
	k	28.946	38.667	51.988	17.262	19	15.22	17.713	14.667
	θ-w	0.04002	0.05799	0.05453	0.0416	0.0147	0.02219	0.02551	0.0323
	Hd	1	1	1	0.998	1	1	1	0.997

*N*, total number of sequences analysed; h, number of haplotypes; s, total number of segregating sites; η (Eta), total number of mutations; π, nt diversity; k, average number of nt differences between sequences; θ-w, Watterson’s estimate of the population mutation rate based on the total number of segregating sites per site (DNA polymorphism); Hd, haplotype diversity.

*The highest value in a category of a particular population type.

†The lowest value in a category of a population type.

Our results indicated high pairwise nt diversity in global TSWV sequences. Acknowledging the tenfold elevated π value of global than local TSWV population sets indicated the spatial conservation of the N gene. Overall, the N gene was highly conserved followed by NSs, and the least conserved was the NSm gene. This could be related to the role of the NSm gene as an effector [19] or symptom development or cell-to-cell movement, which might subject it to different selection pressures. The π values obtained for TSWV peanut isolates in this study were aligned with TSWV peanut population sets from NC, VA [50] and GA [51]. This suggested an equivalent diversity of N, NSm and NSs genes in the peanut crop from year 1997 to 2024. It implied low levels of heterogeneity due to well-mixed and stable virus population with similar selective pressures in different regions of the USA. This was also evident in phylogenetic analysis where N, NSm and NSs gene sequences from TSWV peanut isolates from southeastern states of the USA shared the clades with TSWV-GA isolates. On comparing genes, NSm gene tended to have the highest genetic diversity, followed by NSs gene, and the most conserved gene was the N gene, which has been supported by other studies [21,47]. This did indicate a subtle difference in nt diversity amidst the selected genes in different crops, suggesting an overall similarity with host-based fine beneficial variations between TSWV isolates from peanut, pepper, tobacco and tomato crops. Further investigation is needed to assess host-influenced variations in the virus genome.

### High non-synonymous substitution rate in NSs gene of TSWV isolates

Estimation of selection pressure (dN/dS) on TSWV genomic components suggested a purifying selection due to higher numbers of synonymous mutations. TSWV tobacco isolates that encoded NSs gene sequences contained the highest non-synonymous substitution in both local and global population sets (Table 3c). However, its N gene sequences possessed the least non-synonymous substitutions (only in the local population set) (Table 3a). In local populations, TSWV tomato isolates exhibited the highest non-synonymous substitutions in the N gene, while tobacco isolates also showed maximum substitutions in the NSm gene (Table 3b, c). Contrastingly, TSWV-GA isolates demonstrated 2 to 6 times higher *ω* values for NSm and 3 to 14 times higher *ω* values for NSs genes (Table 3c) in comparison with N gene (for simplicity, N<NSm<NSs). Our analysis suggested that the N gene had approximately a 50% lower rate of non-synonymous substitution in comparison with NSm. In contrast, NSm showed a lower rate (38.8%) of non-synonymous substitution on comparing with NSs gene. Therefore, this indicated a strong purifying selection pressure on the TSWV N gene. Similar results were also obtained in the global N gene sequences including Turkish isolates obtained from tomato, pepper and *Chenopodium album* [22].

**Table 3.** Estimation of selection pressure based on the ratio of non-synonymous substitution to synonymous substitution mutations in the N, NSm and NSs genes of TSWV

TSWV gene	Parameters used	Global population (*n*=178)				Local population (*n*=94)			
		Pepper	Tomato	Tobacco	Peanut	Pepper	Tomato	Tobacco	Peanut
**(a) N**	NonSyn sites	608.6	608.41	608.36	608.04	608.17	608.04	608.18	608.02
	Syn sites	165.4	165.59	165.64	165.96	165.83	165.96	165.82	165.98
	dN; Pi(a)	0.0073	0.00644	0.00625	0.00215	0.00172	0.00181	0.00105	0.00134
	dS; Pi(s)	0.09073	0.08423	0.10404	0.04921	0.03503	0.03346	0.03974	0.0418
	Omega (ω)	0.0804*	0.0764	0.06	0.0436†	0.0491	0.054*	0.0264†	0.032

		Global population (*n*=187)				Local population (*n*=111)			
**(b) NSm**	NonSyn sites	710.59	710.36	710.85	710.52	711.65	710.91	711.17	710.51
	Syn sites	195.41	195.64	195.15	195.48	194.35	195.09	194.83	195.49
	dN; Pi(a)	0.00963	0.00917	0.01003	0.0042	0.00835	0.00494	0.00713	0.00376
	dS; Pi(s)	0.11203	0.1131	0.11423	0.05013	0.03982	0.05345	0.05521	0.04473
	Omega (ω)	0.0859	**0.081†**	0.0878*	0.0837	0.2096*	0.0924	0.1291	**0.084†**

		Global population (*n*=161)				Local population (*n*=78)			
**(c) NSs**	NonSyn sites	1085.62	1085.6	1085.4	1073.36	1085.67	1085.78	1086.14	1073.38
	Syn sites	312.38	312.4	312.6	309.64	312.33	312.22	311.86	309.62
	dN; Pi(a)	0.00992	0.01158	0.01767	0.00541	0.00722	0.00475	0.00809	0.00462
	dS; Pi(s)	0.06509	0.08248	0.1031	0.03655	0.03574	0.03222	0.02825	0.03103
	Omega (ω)	0.1524	**0.1403†**	0.1713*	0.148	0.202015	**0.147424†**	0.286372*	0.148888

NonSyn, non-synonymous substitution mutation; Syn, synonymous substitution mutation; dN, rate of non-synonymous substitution mutation; dS, rate of synonymous substitution mutation; *ω*, omega (dN/dS ratio).

*The highest value in a category of a particular population type.

†The lowest value in a category of a population type.

Overall, the selection pressure deduced from the ratio of non-synonymous substitution to synonymous substitution (*ω*<1) in N, NSm and NSs genes of TSWV isolates implied the presence of purifying selection. This indicated the selection of TSWV isolates with lesser substitution in the aa composition of proteins encoded by the selected genes. Lower *ω* values for the N gene depicted the high importance of N protein in all the crops studied. NSs gene was subjected to lower purifying selection based on the aa substitution rate, which could be co-related with silencing suppressor activity of its product.

### Significant negative selection pressure on most of the codons of N, NSm and NSs genes

SLAC selection tests suggested selection pressures on several codons of N, NSm and NSs genes of TSWV isolates obtained from peanut, pepper, tobacco and tomato. The analysis of the N gene obtained from TSWV-US isolates indicated significant (*P*-value<0.2) negative selection on 16 codons (TSWV peanut isolates), 4 codons (TSWV pepper isolates), 9 codons (TSWV pepper isolates) and 11 codons (TSWV tomato isolates). Collectively, 43 codons of the N gene showed significant negative selection. Further, 39 codons of the N gene of TSWV-GA isolates encountered negative selection pressure. However, fewer codons were subjected to significant positive selection including tenth codon of N gene (Table S3). In the NSm gene, 17 codons (TSWV peanut isolates), 8 codons (TSWV pepper isolates), 22 codons (TSWV tobacco isolates) and 28 codons (TSWV tomato isolates) experienced negative selection. Similarly, negative selection in NSm gene was observed on 19 codons in TSWV-GA peanut, 8 codons in TSWV-GA pepper, 10 codons in TSWV-GA tobacco and 22 codons in TSWV-GA tomato isolates. Further, in NSs gene (TSWV-US), negative selection was significant on 19 codons (TSWV peanut isolates), 8 codons (TSWV pepper isolates), 10 codons (TSWV tobacco isolates) and 22 codons (TSWV tomato isolates) in different crop hosts.

In this investigation, negative selection was significant for each of the evaluated genes. The significant positive selections were observed at tenth and fifteenth codons of the NSm gene of TSWV-GA isolates where serine (polar and uncharged aa) was replaced by leucine/valine (non-polar and uncharged aa) (Table S3). This indicated a 50% chance of conversion of polar aa to a non-polar aa, which could lead to functional changes. Although the initial 22 aa make a disordered protein structure (https://www.uniprot.org/uniprotkb/P36292), important residues for movement function were reported at different positions in the NSm protein of TSWV. Further alignment of the NSm protein of TSWV-GA isolates also revealed the conversion of alanine to threonine (polar and uncharged aa) at fifty-second and serine to leucine at two hundred seventy-seventh position in all TSWV-GA sequences. These mutations lie in the region important for tubule formation and cell-to-cell movement [6]. Similarly, the significant positive selection at four hundred thirty-seventh codon of NSs was observed where serine was converted to phenylalanine (non-polar and uncharged). This position lies in the C-terminal (NSs protein), which most likely plays a structural role [7]. This indicated overall low positive selection on codons of all three genes. Therefore, an inference could be drawn that most codons were under negative selection with a few codons under positive selection, suggesting overall purifying selection. This also coincided with the results of different analyses conducted in this study. The global TSWV peanut isolates had the greatest number of codons under negative selection, which overlaps with the genetic diversity results where global peanut isolates were most conserved. Also, other mutations were observed in all the three genes, and further analysis is required to make inferences (Table S4 and Fig. S3).

### Intense purifying selection on N, NSm and NSs genes of TSWV isolates from selected crops

Fu and Li’s D* statistic compared the number of singleton mutations to the total number of mutations, whereas Fu and Li’s F* statistic compared the number of singleton mutations to the average number of nt differences between the pairs of sequences. The test statistics for global and local populations of TSWV isolates were significant negative values (varies from −1 to −5), indicating a non-neutral evolution. The D* statistics of the global population of TSWV NSs gene had a lower negative value than NSm and N genes, indicating stronger purifying selection on NSs- gene due to the presence of an excess of rare variants. In TSWV-GA isolates, the N gene identified in tobacco had the significant D* value (D*=−2.619, *P*-value<0.01) and F* value (F*=−2.83115, *P*-value<0.01) with Tajima’s D stats (Tajima’s D=−1.96146, *P*-value<0.01), whereas in the global scenario, TSWV isolated from peanut had statistically significant values of F* (F*=−2.42646, *P*-value<0.01) with Tajima’s D stats (Tajima’s D=−1.96566, *P*-value<0.01) (Table 4a), indicating significant negative selection. Based on NSm gene sequences, test statistics had the lowest significant negative values for TSWV-global peanut isolates. However, TSWV-GA peanut isolates showed ~17% higher significant negative values from TSWV global peanut isolates (Table 4b). Analogously, global TSWV peanut isolates displayed the lowest significant negative values established on NSs gene (Table 4c). In summary, significant statistical values indicated that the observed deviations were unlikely to be due to random chance. The Z scores were shown to compare the test statistics with variable sample sizes of different population sets.

**Table 4.** Statistical results from neutrality tests (Fu and Li’s F* and D* and Tajima’s D statistics) to evaluate demographic changes in N, NSm and NSs genes of TSWV

TSWV gene	Parameters used	Global population (*n*=178)				Local population (*n*=94)			
		Pepper	Tomato	Tobacco	Peanut	Pepper	Tomato	Tobacco	Peanut
**(a) N**	D*	−1.71363	−1.8023	−2.0876	−2.06258	−0.70777	−1.77445	−2.619*	−1.41679
		(NS)	(NS)	(NS)	(NS)	(NS)	(NS)	(*P*<0.01)	(NS)
	**Z score**	0.366778	0.278108	−0.00719	0.017828	1.372638	0.305958	−0.53859	0.663618
	F*	−1.81137	−1.99983	−1.9631	−2.42646*	−0.76532	−1.96587	−2.83115*	−1.77436
		(NS)	(NS)	(NS)	(*P*<0.01)	(NS)	(NS)	(*P*<0.01)	(NS)
	**Z score**	1.076722	0.888262	0.924992	0.461632	2.122772	0.922222	0.056942	1.113732
	**Tajima's D**	−1.17389	−1.45605	−0.89117	−1.96566*	−0.64848	−1.49048	−1.96146*	−1.61987
		(NS)	(NS)	(NS)	(*P*<0.01)	(NS)	(NS)	(*P*<0.01)	(NS)
	**Z score**	2.031007	1.748847	2.313727	1.239237	2.556417	1.714417	1.243437	1.585027

		Global population (*n*=187)				Local population (*n*=111)			
**(b) NSm**	D*	−0.90654	−2.33087	−2.63261*	−3.39041**	−1.06629	−2.55218*	−2.42329	−2.75005*
		(NS)	(NS)	(*P*<0.01)	(*P*<0.05)	(NS)	(*P*<0.01)	(NS)	(*P*<0.01)
	**Z score**	1.997647	0.573317	0.271577	−0.48622	1.837897	0.352007	0.480897	0.154137
	F*	−1.16599	−2.31745	−2.46634*	−3.44516**	−1.17904	−2.62498*	−2.4729	−2.90945*
		(NS)	(NS)	(*P*<0.01)	(*P*<0.05)	(NS)	(*P*<0.01)	(NS)	(*P*<0.01)
	**Z score**	2.113218	0.961758	0.812868	−0.16595	2.100168	0.654228	0.806308	0.369758
	**Tajima's D**	−1.1409	−1.3342	−1.14146	−1.98762*	−0.94394	−1.55252	−1.43777	−1.88263*
		(NS)	(NS)	(NS)	(*P*<0.01)	(NS)	(NS)	(NS)	(*P*<0.01)
	**Z score**	2.976531	2.783231	2.975971	2.129811	3.173491	2.564911	2.679661	2.234801

		Global population (*n*=161)				Local population (*n*=78)			
**(c) NSs**	D*	−2.4357	−3.1404*	−2.636*	−4.9257**	−0.6073	−2.6194*	−2.7874**	−4.61781**
		(NS)	(*P*<0.01)	(*P*<0.01)	(*P*<0.05)	(NS)	(*P*<0.01)	(*P*<0.05)	(*P*<0.05)
	**Z score**	−0.15941	−0.86418	−0.35972	−2.64948	1.668943	−0.34314	−0.51117	−2.34157
	F*	**−2.5913**	−3.1236*	−2.4791	−4.8156**	−0.6411	−2.846*	−2.9821**	−4.22239**
		(NS)	(*P*<0.01)	(NS)	(*P*<0.05)	(NS)	(*P*<0.01)	(*P*<0.05)	(*P*<0.05)
	**Z score**	−0.08955	−0.62191	0.022595	−2.31387	1.860645	−0.34429	−0.48039	−1.72069
	**Tajima's D**	−1.70129	−1.63035	−0.88338	−2.48131**	−0.6073	−2.02954*	−2.04396*	−2.41776**
		(NS)	(NS)	(NS)	(*P*<0.05)	(NS)	(*P*<0.01)	(*P*<0.01)	(*P*<0.05)
	**Z score**	1.097619	1.168559	1.915529	0.317599	2.191609	0.769369	0.754949	0.381149

D*, Fu and Li’s D* test statistics; F*, Fu and Li’s F* test statistics; Tajima’s D, Tajima’s D test statistics. PM test: probability obtained by the permutation test with 1,000 replicates, ns, not significant; *, 0.01<P<0.05; **, 0.001<P<0.01.

The NSm and NSs genes of TSWV peanut isolates in both the population groups displayed the highest purifying selection in comparison with other population sets. The N gene in TSWV global peanut isolates was undergoing maximum negative selection. Shift in the cultivation of peanut variety from Florunner to Georgia Green (1997) might have played a role in providing selection pressure for certain TSWV peanut isolates. However, the N gene in TSWV tobacco isolates was under the most negative selection in the local population set. Our study demonstrated that the evolution of TSWV was deviated from the neutral theory of molecular evolution. These tests suggested that natural selection and demographic events (like population bottlenecks such as monoculture of cultivars with qualitative resistance in pepper and tomato, and cultivars with moderate resistance in peanut or founder effect) have influenced the genetic makeup of the TSWV population in peanut, pepper, tobacco and tomato crops. This was also evident in other studies conducted using TSWV isolates as well as other viruses prevalent in this region [52] irrespective of the crop [22,53].

### High gene flow and minimal genetic differentiation among crop-based TSWV isolates

Population accumulates genetic differences with time, often due to limited gene flow and different selective pressures. Estimates like Fst, Dxy, Hs, Ks*, Kst*, Z*, Snn and Nm were analysed to measure genetic flow and differentiation. The Hs or haplotype-based statistics (representative of average gene diversity within a subpopulation) value for NSs gene was highest (Hs=1) in both global and local populations of TSWV isolates. This indicated high diversity in NSs gene across the globe including GA. Among the global samples, comparisons between TSWV tomato and TSWV peanut isolates displayed low Dxy estimate for the selected genes. In contrast, the comparison between TSWV tobacco and TSWV pepper isolates exhibited a high Dxy estimate in the same selected genes. However, the comparison between TSWV pepper and TSWV peanut isolates displayed high Dxy estimate for N and NSm genes in the local population sets as well (Table 5a, b). Contrastingly, the Dxy estimate was lowest for the above-mentioned comparison based on NSs gene (Table 5c).

**Table 5.** Gene flow and genetic differentiation estimates between the population sets of N, NSm and NSs genes of TSWV

TSWV gene	Population-I	Population-II	Global (*n*=178)			Local (*n*=94)		
			Hs	Dxy	Fst	Hs	Dxy	Fst
**(a) N**	Tomato	Tobacco	0.99756	0.02587	0.02749	0.99745	0.00959	0.06259
	Tomato	Pepper	0.99923	0.02419	**0.00609†**	0.99548	0.00886†	0.05497
	Tomato	Peanut	0.99496	**0.01906†**	0.07538	0.99154	0.00991	0.06448*
	Tobacco	Pepper	0.99753	0.02728*	0.04149	1	0.00941	**0.03080†**
	Tobacco	Peanut	0.99418	0.02196	0.10166	0.99376	0.01026*	0.05497
	Pepper	Peanut	0.99432	0.02216	0.16038*	0.9915	0.00982	0.04307

			Global (*n*=187)			Local (*n*=111)		
**(b) NSm**	Tomato	Tobacco	0.99521	0.03279	0.0242	0.98632	0.0176	0.06946
	Tomato	Pepper	0.99834	0.03238	**0.02264†**	0.99281	0.01988	0.23582
	Tomato	Peanut	0.99744	**0.02539†**	0.10057	0.99372	**0.01455†**	**0.04128†**
	Tobacco	Pepper	0.995	0.03245*	0.01281	0.9871	0.01747	0.07062
	Tobacco	Peanut	0.99429	0.02556	0.09157	0.98992	0.01719	0.12823
	Pepper	Peanut	0.9984	0.02566	0.10862*	0.99742	0.02021*	0.31709*

			Global (*n*=161)			Local (*n*=78)		
**(c) NSs**	Tomato	Tobacco	1	0.03377	0.06084	1	0.03377	0.06084
	Tomato	Pepper	1	0.02549	**0.03297†**	1	0.02549	**0.03297†**
	Tomato	Peanut	0.999	**0.02234†**	0.11559	0.999	0.02234	0.11559
	Tobacco	Pepper	1	0.03411*	0.14234	1	0.03411*	0.14234
	Tobacco	Peanut	0.99873	0.03043	0.19948	0.99873	0.03043	0.19948
	Pepper	Peanut	0.99863	0.02228	0.22368*	0.99863	**0.02228†**	0.22368*

Hs, haplotype-based statistics; Fst, nt sequence-based statistics where Fst values were used to measure the extent of gene flow; Dxy, average nt differences between the populations.

*The highest value in a category of a particular population type.

†The lowest value in a category of a population type.

Fixation index (Fst) indicates the genetic differentiation between populations. In global populations of TSWV isolates, the Fst estimate was highest between TSWV pepper and TSWV peanut isolates (for N, NSm and NSs genes) (Table 5a–c). Similarly, TSWV-GA isolates from pepper and peanut comparison had high Fst estimate for NSm and NSs gene (Table 5b, c). However, the Fst value for the N gene was observed to be highest for TSWV tomato and TSWV pepper isolates. nt sequence-based statistics like Ks*, Kst*, Z*, Snn and Nm depict overall scenarios of gene flow and differentiation. Higher values indicate genetic differentiation, and lower values indicate elevated gene flow. For N and NSm genes, the TSWV global population had higher values of Ks*, Z*, Snn and Kst* than the local population (except Kst* for NSm gene) estimates (Table 6). This indicated higher genetic differentiation in isolates reported from different crops, which were dispersed in diverse geolocations.

**Table 6.** Gene flow and genetic differentiation estimates based on N, NSm and NSs genes of TSWV

Parameter used	N		NSm		NSs	
	Local	Global	Local	Global	Local	Global
Ks*	15.85404	2.0353	3.07564	2.57438	3.31972	3.31972
Kst*	0.05576***	0.01655***	0.02234***	0.0414***	0.03712***	0.03712***
Z*	8.54635***	7.32525***	8.60598***	7.48633***	8.28196***	8.28196***
**Snn**	0.49622***	0.49142***	0.52513***	0.53772***	0.53189***	0.53189***
**Nm**	3.56	5.14	4.28	1.38	1.72	1.72

Ks*, Kst*, Z* and Snn represent nt sequence-based statistics. For N gene, chi-square (table), Chi2: 521.165, *P*-value of Chi2: 0.0945* (df=480); for NSm gene, chi-square (table), Chi2: 542.010, *P*-value of Chi2 : 0.2344* (df=519); and for NSs gene, chi-square (table), Chi2: 486.000, *P*-value of Chi2: 0.3779* (df=477). PM test: probability obtained by the permutation test with 1,000 replicates, ns, not significant; *, 0.01<*P*<0.05; **, 0.001<*P*<0.01; ***, *P*<0.00.

This study indicated high gene flow and low genetic differentiation among the TSWV isolates obtained from peanut, pepper, tobacco and tomato crops (0.5>Fst). This supported our hypothesis of intermixing of TSWV isolates between the crops cultivated in proximity. Furthermore, it disapproves the presence of genetic differentiation between TSWV isolates present in row and horticultural crops. Additionally, Hs values close to one for all the comparisons indicated a high genetic diversity in each population. N gene showed comparatively low Fst values, indicating uniformity in N gene sequences across different crops. This was also supported by the Nm values (>1.0) with higher differentiation in the NSm gene. The subtle differences were discovered between global population sets of TSWV isolates from peanut and pepper crops. Similar pattern was also observed in the local TSWV isolate population (except for N gene). Broadly, lower gene flow was observed in the selected genes of TSWV global populations. This supports our previous results of higher genetic diversity among TSWV isolates obtained from different locations. Further, TSWV isolates from different crops could be biologically characterized to understand the subtle difference. Due to the presence of secondary structures in genomic RNAs of TSWV, sequencing whole genome might provide a coherent understanding of changes associated with each host along with passage studies in one crop with isolates collected from another crop host.

### Recent common ancestral origin for selected crop-based TSWV isolates

**Fig. 4.**
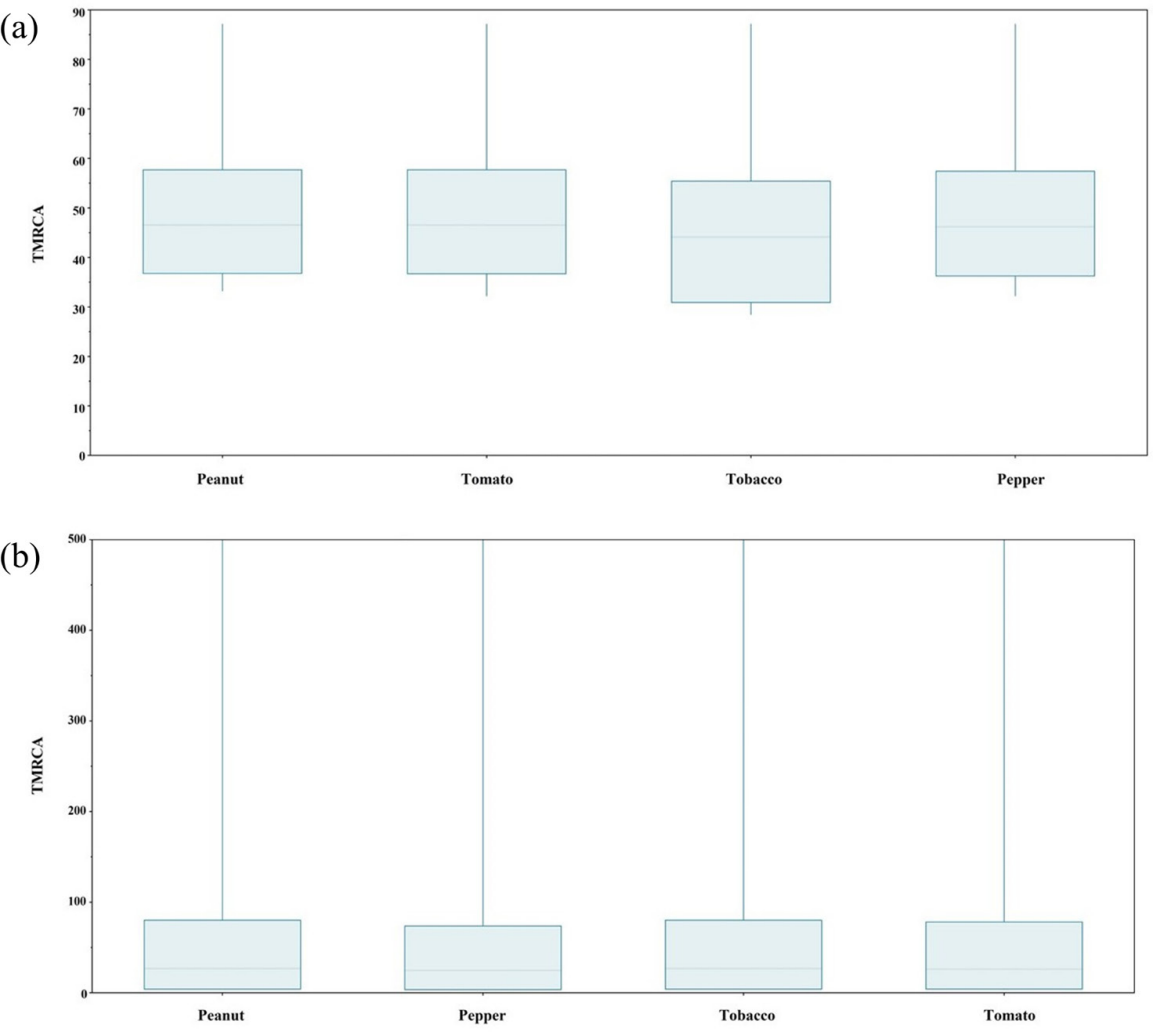
Estimation of TMRCA of N gene of TSWV-US and TSWV-GA. (**a**) Box plot representing the N gene of TSWV-US isolates collected from peanut, pepper and tomato from the year 1997 to 2024 with estimated TMRCA to be ~46 years ago (1977), whereas TMRCA for tobacco isolates was ~44 years ago (1979). (**b**) Box plot representing recent isolates of TSWV-GA from peanut, and tobacco with estimated TMRCA to be ~26 years ago (1997), whereas TMRCA for pepper and tomato was ~24 years ago (1999).

**Fig. 5.**
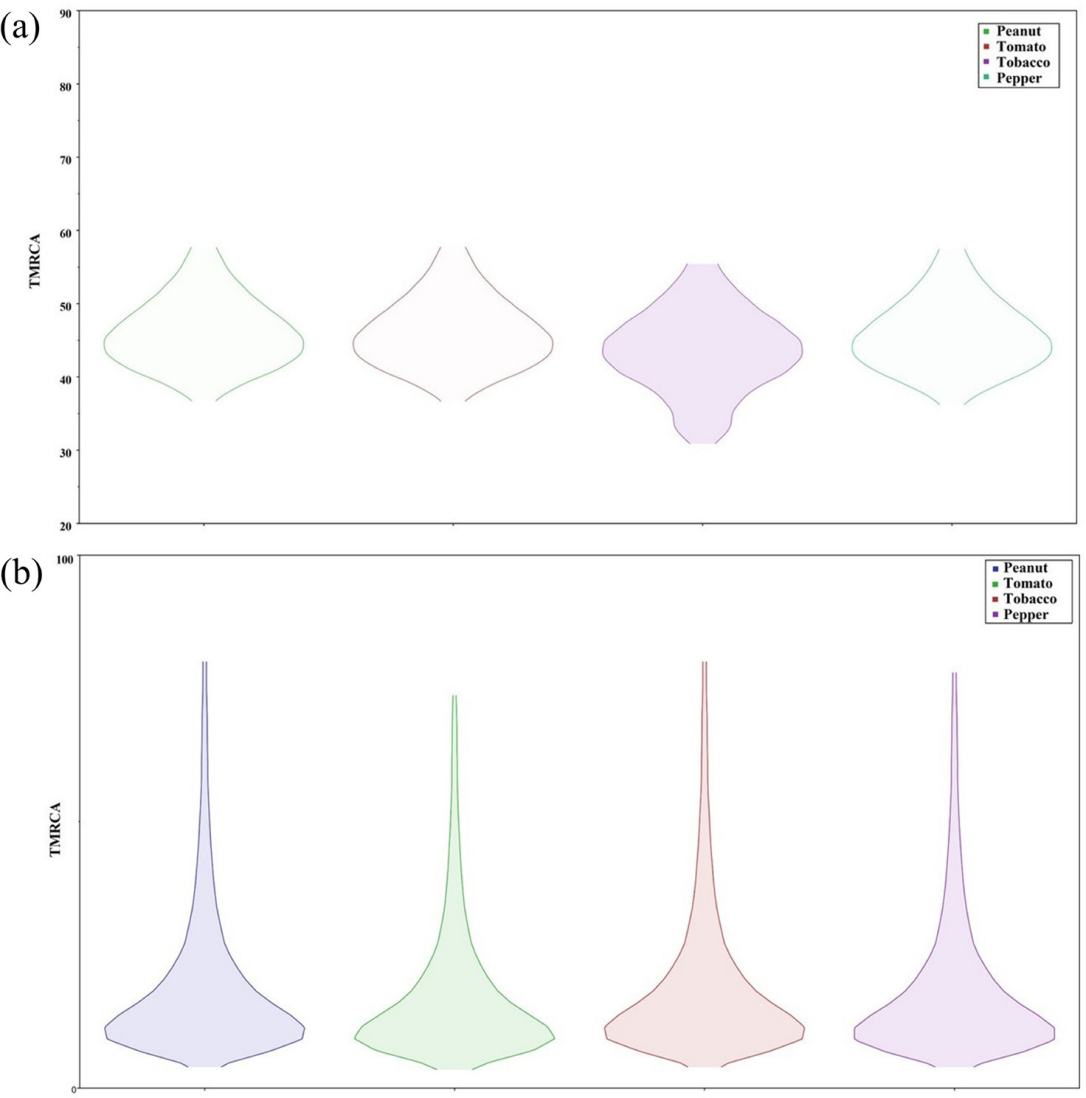
Marginal density plot showing kernel density estimates for TMRCA based on TSWV N gene. (**a**) Violin plot chart represents the data points for TMRCA of TSWV-US isolates collected from peanut, pepper, tobacco and tomato from the year 1997 to 2024, whereas the violin plot in part (**b**) of the figure represents TMRCA of recent isolates of TSWV-GA from peanut, tobacco and tomato. These plots illustrated that the probability mass distribution was around the median for all the cases.

**Fig. 6.**
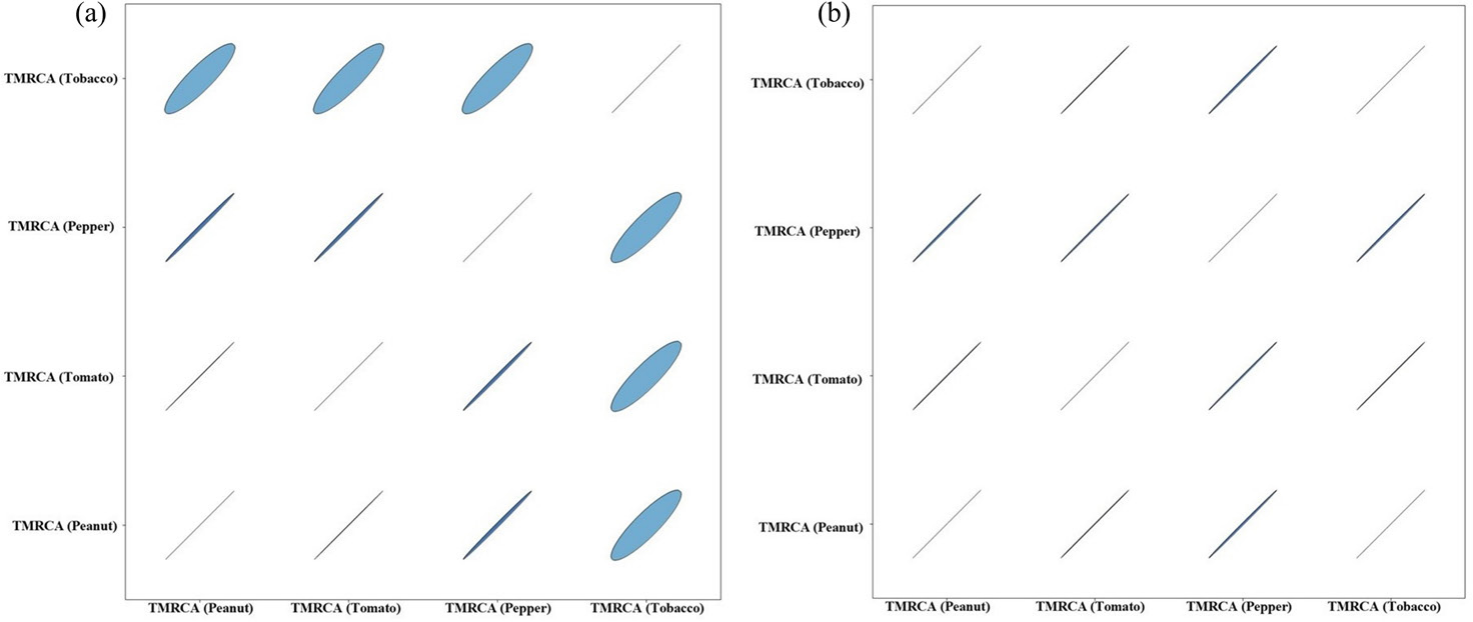
Joint marginal plot for (**a**) TSWV-US and (**b**) TSWV-GA N gene illustrating extension for correlation between the crops. Colour gradients indicate strength and direction of correlation, with shades of blue indicating positive correlation. Shape of ellipse reinforces the strength of correlation, where a circle represents no correlation and a line shows perfect correlation.

Summary statistics were obtained for the time to most recent common ancestor (TMRCA) of N, NSm and NSs genes in peanut, pepper, tobacco and tomato crops from tracer panel (Table S6 and Figs S4, S5 and S6). In our study, we found the TMRCA for TSWV-US (based on N gene sequences from TSWV-US isolates) collected from peanut, pepper and tomato crops from the year 1997 to 2024 was ~46 years ago (1977), whereas in tobacco (Fig. 4a), TMRCA was ~44 years ago (1979). However, for TSWV-GA isolates (from the year 2023–24) from peanut and tobacco, the estimated TMRCA was ~26 years ago (1997), whereas for TSWV pepper and tomato isolates (Fig. 4b), the TMRCA was ~24 years ago (1999). This clearly indicates that the most recent common ancestor of the TSWV-GA population might have originated in the late 1990s. The marginal density plot suggested that N gene-based TMRCA ranged from the year 1966 to 1987 for TSWV-US isolates from (CA, GA, NC, NM, SC, TX and WA) (Fig. 5a), whereas the TMRCA range for TSWV-GA isolates was depicted from 1943 to 2020, suggesting a recent emergence of TSWV isolates in GA (Fig. 5b). Joint marginal plot showed the highest correlation in TMRCA between solanaceous (tomato and pepper) and leguminous (peanut) crops under study (Fig. 6a). In contrast, TSWV-GA isolate-based analysis suggested a high correlation in TMRCA among all the taxa (Fig. 6b). This result suggested a similar evolutionary pressure, or events might have influenced their genetic divergence. The mean substitution rate for N, NSm and NSs gene sequences suggested 4.584×10^−4^ [95% highest posterior density (HPD) with a range of 3.218×10^−4^ to 6.091×10^−4^], 5.371×10^−4^ (95% HPD with a range of 4.083×10^−4^ to 6.658×10^−4^) and 4.906×10^−4^ (95% HPD with a range of 4.083×10^−4^ to 6.658×10^−4^) substitution per site per year, respectively, whereas alpha values for N, NSm and NSs genes were 6.604×10^−2^, 19.9×10^−2^ and 36.6×10^−2^, respectively. This indicated that the rate of heterogeneity was higher in N gene as compared with NSm or NSs gene.

Our investigation indicated that the most common recent ancestor of TSWV peanut and tobacco isolates was likely in the 1970s. This coincided with the time of first detection of TSWV in the US peanut crop [54]. TSWV took a decade to cause significant disease pressure in tobacco (first reported in the late 1980s) [55], pepper and tomato (in the 1980s) [56] crops. This can be attributed to the expansion of thrips species to the southeast part of the USA in the late 1980s. Furthermore, the TMRCA for recent TSWV-GA populations dates to 1997 root-age, which can be highly correlated to our recorded data of DI in commercially grown peanut where the highest TSWD incidence was evaluated in the year of 1997. This might have contributed to an increase in the diversity of TSWV quasispecies. In due course of time, TSWV isolates adapted to moderately resistant [11] or resistant varieties of different crops might have flourished and led to the abundance of the current TSWV population in southeast USA. According to previous studies, a co-evolutionary dynamic determining transmission efficiency within a single vector species was observed [57]. This indicates the role of vector species in TSWV diversity. Further vector species dynamics along with host might have played a role in determining the prevalent TSWV population. As per our findings, it took nearly 20–30 years to shift from one TSWV ancestral population (1970s) to the next ancestral population (1990s). Consequently, in the next 20–30 years from the 1990s (between the 2020s and 2030s), a shift of TSWV populations could be measured in commercially grown row and horticultural crops in GA, USA. Moreover, studying the TSWV diversity in thrips would provide crucial insights about the isolates selected in the overall agro-ecosystem.

## Conclusion

The remarkable adaptability of TSWV could be attributed to its RNA genome. As a result, changes in the TSWV genome have made resistant varieties non-functional in controlling TSWD in pepper and tomato [17,58]. In addition, the DI remained variable across decades in row crops (peanut and tobacco) despite extensive integrated management practices. Extrapolating the TSWV diversity in peanut, pepper, tobacco and tomato crops indicated an overall similarity between the isolates and lack of host-based adaptation in a specified geographical region. This suggests that the mutations in TSWV have positive pleiotropic effects between hosts as stated in other studies [2]. Higher gene flow between solanaceous crops (pepper and tomato) indicated similar selection pressure in phylogenetically closer hosts. In this study, the N gene was the most conserved, followed by the NSm and NSs genes between the hosts. This could be attributed to the necessity of adjustment in movement [2] and suppression of the RNA silencing mechanism [59] according to the host. TSWV isolates have undergone purifying selection to maintain the crucial functions and mutations with positive pleiotropy in N, NSm and NSs genes. Furthermore, the impact of management strategies could be inferred due to the projection of time to the most common recent ancestors of the current TSWV population without implementation of integrated disease management strategies. Selection of better adapted TSWV isolates across the major crops could be inferred. In summary, our findings suggested intermixing of TSWV isolates between peanut, pepper, tobacco and tomato crops, which could be due to the presence of isolates performing better in each crop and vector species simultaneously. Factors supporting the abundance of positive pleiotropic mutations would be overlapping of cultivation season, field proximity and the same vector species’ population transmitting TSWV between crops. This study was formulated to analyse host effects on TSWV isolates as the virus needs to adapt to a new host. Instead, the presence of a large single population of TSWV adapted to a geographical region with positive pleiotropy was inferred. In summary, TSWV evolution could be speculated to be tailored according to a region, and high TSWD incidence generated enough raw material for selection pressure to act on it.

## Supplementary material

10.1099/jgv.0.002119 Supplementary Material 1.

## Abbreviations:

BEAST: Bayesian Evolutionary Analysis Sampling Trees
C-terminal: carboxy terminal
DI: disease incidence
dN: rate of non-synonymous substitution mutation
dS: rate of synonymous substitution mutation
h: haplotype number
Hd: haplotype diversity
HPD: highest posterior density
iTOL: Interactive Tree of Life
k: average number of nt differences
L RNA: large RNA
MCMC: Markov chain Monte Carlo
ML: maximum likelihood
M RNA: medium RNA
N: nucleocapsid
NSm: nonstructural movement
NSs: nonstructural silencing suppressor
N-terminal: amino terminal
RB: resistance breaking
S: segregating site
SLAC: single-likelihood ancestor counting
S RNA: small RNA
TMRCA: time to most recent common ancestor
TSWD: tomato spotted wilt disease
TSWV: tomato spotted wilt virus
TSWV-GA: TSWV-Georgia
UGA: University of Georgia
η (Eta): total number of mutations
θ-w: Watterson’s theta
π: average nt diversity
*ω*: omega (dN/dS ratio)
