# Supplementary material for: Molecular genetic analyses of the N, NSm and NSs genes of a local population of Orthotospovirus tomatomaculae reveal purifying selection in crops in the southeastern USA
Source: J Gen Virol. 2025 Jul 7;106(7):002119. doi: 10.1099/jgv.0.002119 (PMC12282330; doi:10.1099/jgv.0.002119)
Supplement: Supplementary Material 1. [file jgv-106-02119-s001.pdf]

Supplementary Figures

Fig. S1. Amplification of N-, NSm-, and NSs-gene of **(a)** TSWV pepper isolate for Sanger sequencing. 1% agarose gel with ~750 bp NSs_1 (lane 1-3&5), ~1200 bp NSm (lane 4 &7), ~700 bp NSs_2 (lane 6, &8-10), ~900 bp NSs_3 (lane11-13, & 15), and ~900 bp N (lane16, 17,19, & 20) amplicons with 1kb plus ladder in 14th and 18th lane (14 & 18). **(b)** TSWV tomato isolates for Sanger sequencing. 1% agarose gel with ~750 bp NSs_1 (lane 5), ~1200 bp NSm (lane 15-17), ~700 bp NSs_2 (lane7-10), ~900 bp NSs_3 (lane2-4), and ~900 bp N (lane11-13) amplicons with 1kb plus ladder in 1st, 6th, and 14th lane (1, 6, & 14). **(c)** TSWV tobacco isolates for Sanger sequencing. 1% agarose gel with ~750 bp NSs_1 (lane 7-11), ~1200 bp NSm (lane17-19), ~700 bp NSs_2 (lane 12-16), ~900 bp NSs_3 (lane1-5), and ~900 bp N (lane 21-24) amplicons with 1kb plus ladder in 6th and 20th lane (6 and 20). **(d)** TSWV peanut isolate for Sanger sequencing. 1% agarose gel with ~750 bp NSs_1 (lane 5-7), ~1200 bp NSm (lane 3), ~700 bp NSs_2 (lane 8-10), ~900 bp NSs_3 (lane11-13), and ~900 bp N (lane 11-13) amplicons with 1kb plus ladder in 1st lane (lane 1). The fragments in the orange and blue box are 1500 bp and 500 bp, respectively. 20µl of PCR product was added into each well to further elute the amplicons for sequencing.

Fig. S2. Sequence demarcation tool map of **(a)** N, **(b)** NSm, and **(c)** NSs gene of tomato spotted wilt virus. This map shows the results of pairwise nucleotide identity between all the sequences utilized in this study. Different colors represent percentage of nucleotide identity with red color represents 100% identity and blue color represents 94% (N gene), 91% (NSm gene), and 93% (NSs gene). Sequence demarcation analysis was performed using the Sequence Demarcation Tool (SDT) v1.3.

Fig. S3. Amino acid sequence alignment of **(a)** nucleocapsid protein (encoded by N gene), **(b)** non-structural movement protein (encoded by NSm gene), and **(c)** non-structural silencing suppressor protein (encoded by NSs gene) deduced from gene sequences of TSWV-GA isolates, with reference sequence at the top. Mutations are highlighted with a box.

Fig. S4. Summary statistics for the time to most recent common ancestor (TMRCA) of **(a)** TSWV-NSm and **(b)** TSWV-NSs gene using BEAST analysis. The TMRCA of NSm gene sequences of TSWV isolates from tomato and pepper was ~64 years ago (1959) whereas for TSWV isolates from peanut was ~47 years ago (1976). The latest evolved group of NSm sequences was obtained from TSWV isolates from tobacco, with TMRCA of ~43 years ago (1980). TMRCA based on NSs-gene of TSWV isolates from tomato and pepper was ~44 years ago (1979), followed by TSWV isolates from peanut was ~43 years ago (1980) and lastly TSWV isolates from tobacco was ~28 years ago (1995).

Fig. S5. Marginal density plot showing kernel density estimates for time to most common recent ancestor (TMRCA). **(a)** Violin plot chart represents the data points for TMRCA of tomato spotted wilt virus of US isolates collected from peanut, pepper, tobacco, and tomato from the year 1997 to 2024 based on NSm-gene of TSWV-US isolates. Whereas the violin plot in part **(b)** of the figure represents TMRCA of recent isolates of TSWV-US from peanut, tobacco, and tomato based on NSs-gene. These plots illustrated that the probability mass distribution was around the median for all the cases.

Fig. S6. Joint marginal plot for **(a)** TSWV-NSm and **(b)** NSs gene illustrating extension for correlation between crops. Color gradients indicate strength and direction of correlation with shades of blue indicates positive correlation and shades of red indicates strong negative selection. Shape of ellipse reinforce the strength of correlation, where circle represent no correlation, and a line shows perfect correlation. Yellow colored circle indicates no correlation. The highest correlation in TMRCA of NSm, and NSs sequence from tomato and pepper isolates was observed. Least correlation was seen between TMRCA of NSs sequence from tomato and peanut isolates. This suggested the same evolutionary pressures (host) or events that have influenced their divergence times in a similar manner.

Fig S1a

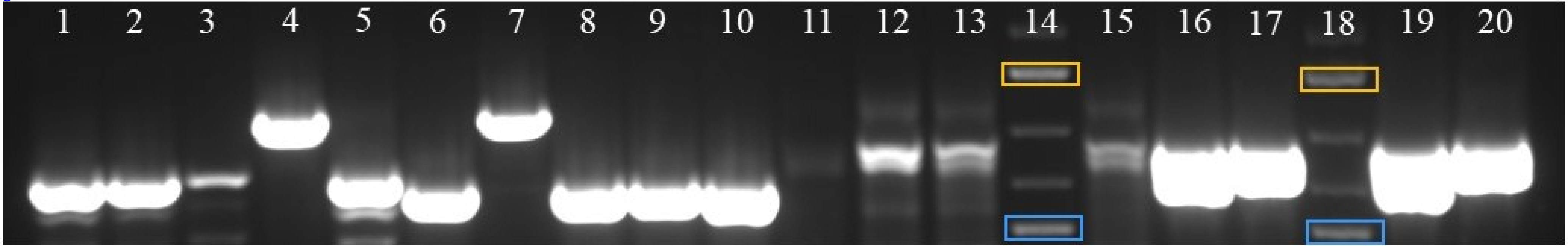

Fig S1b

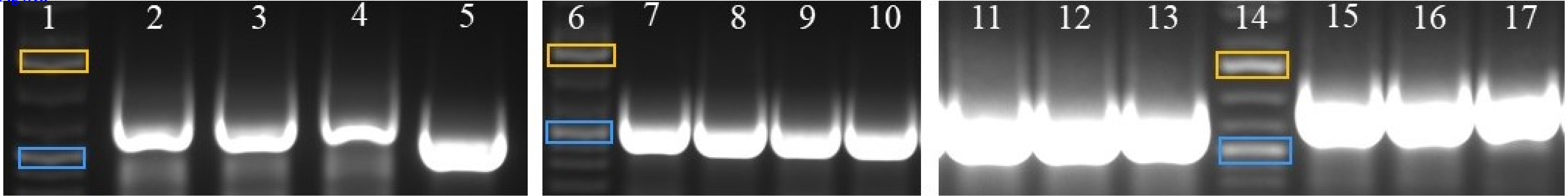

Fig S1c

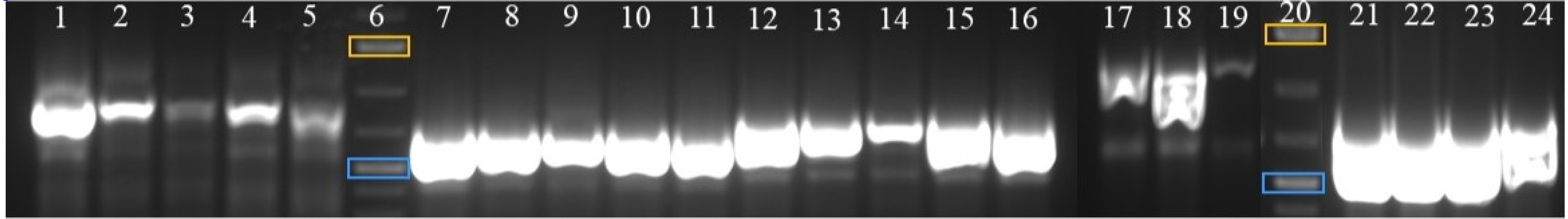

Fig S1d

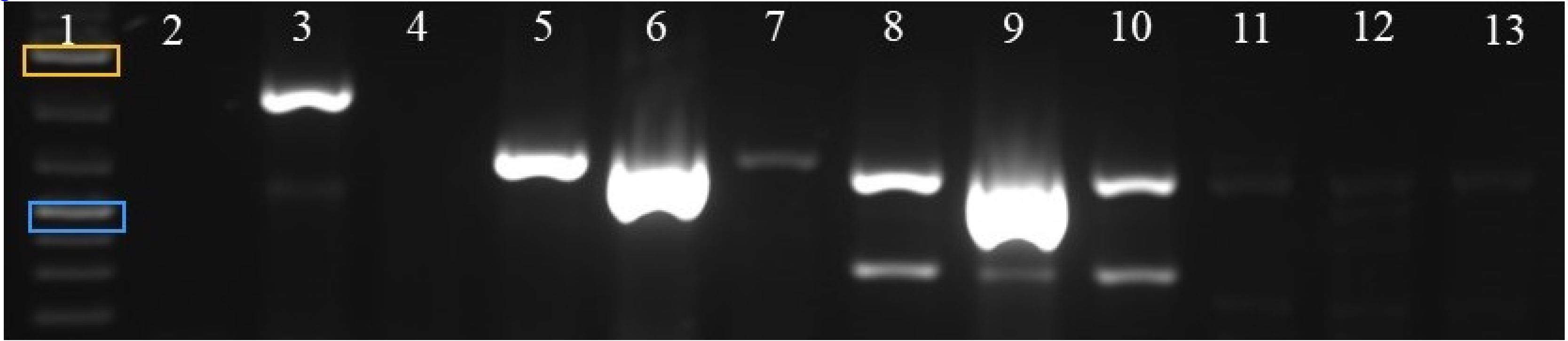

Fig S2a

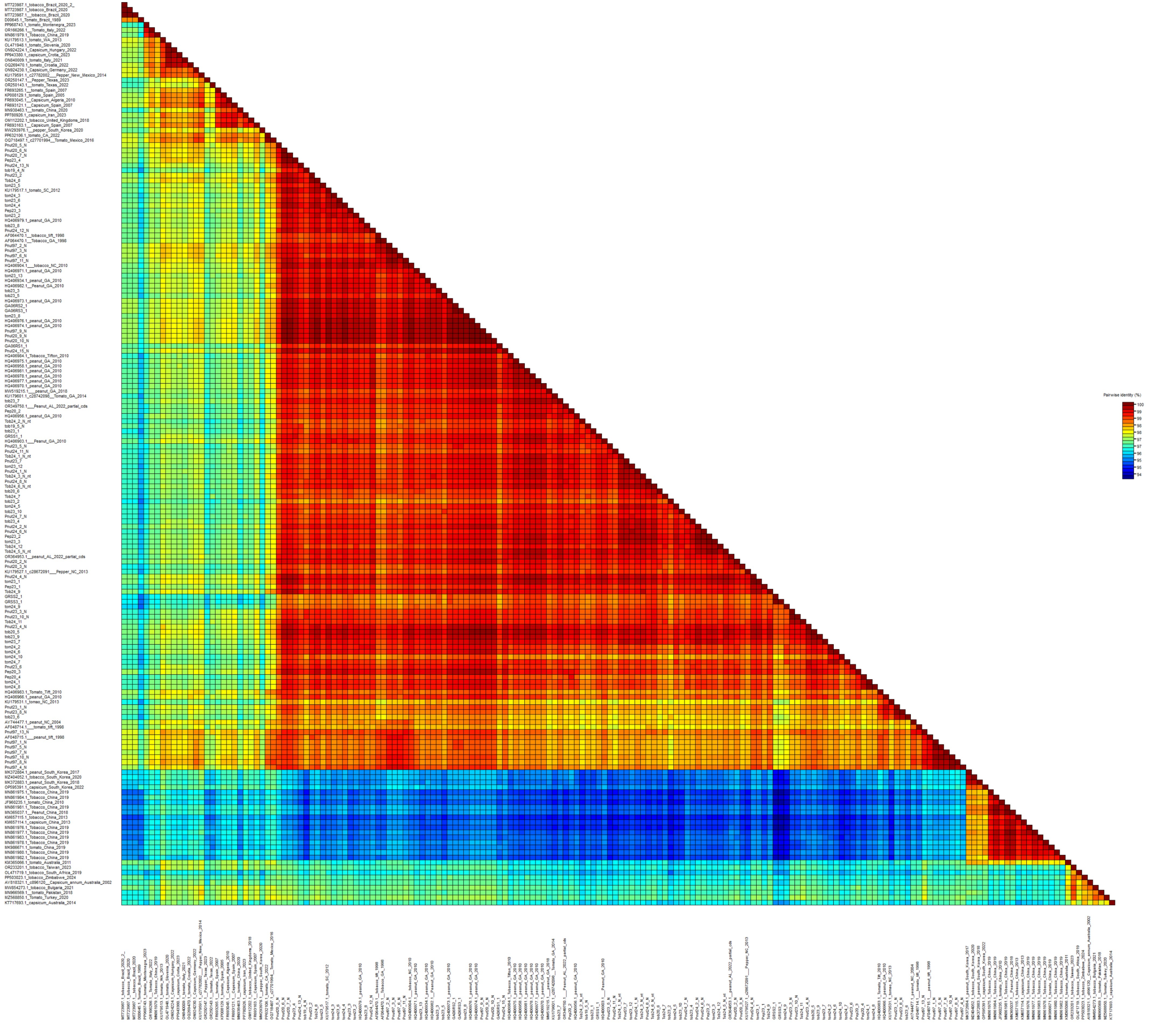

Fig S2b

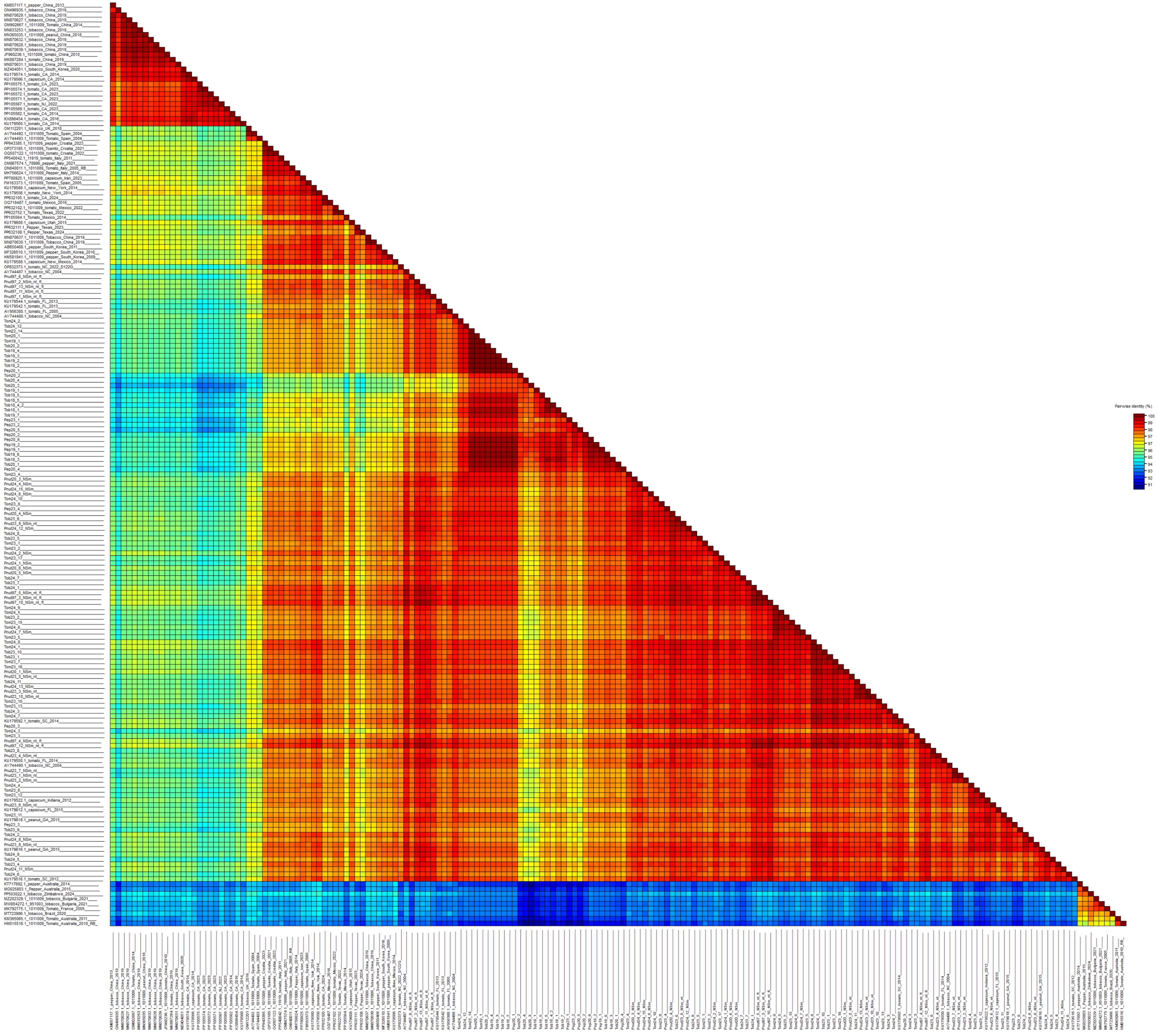

Fig S2c

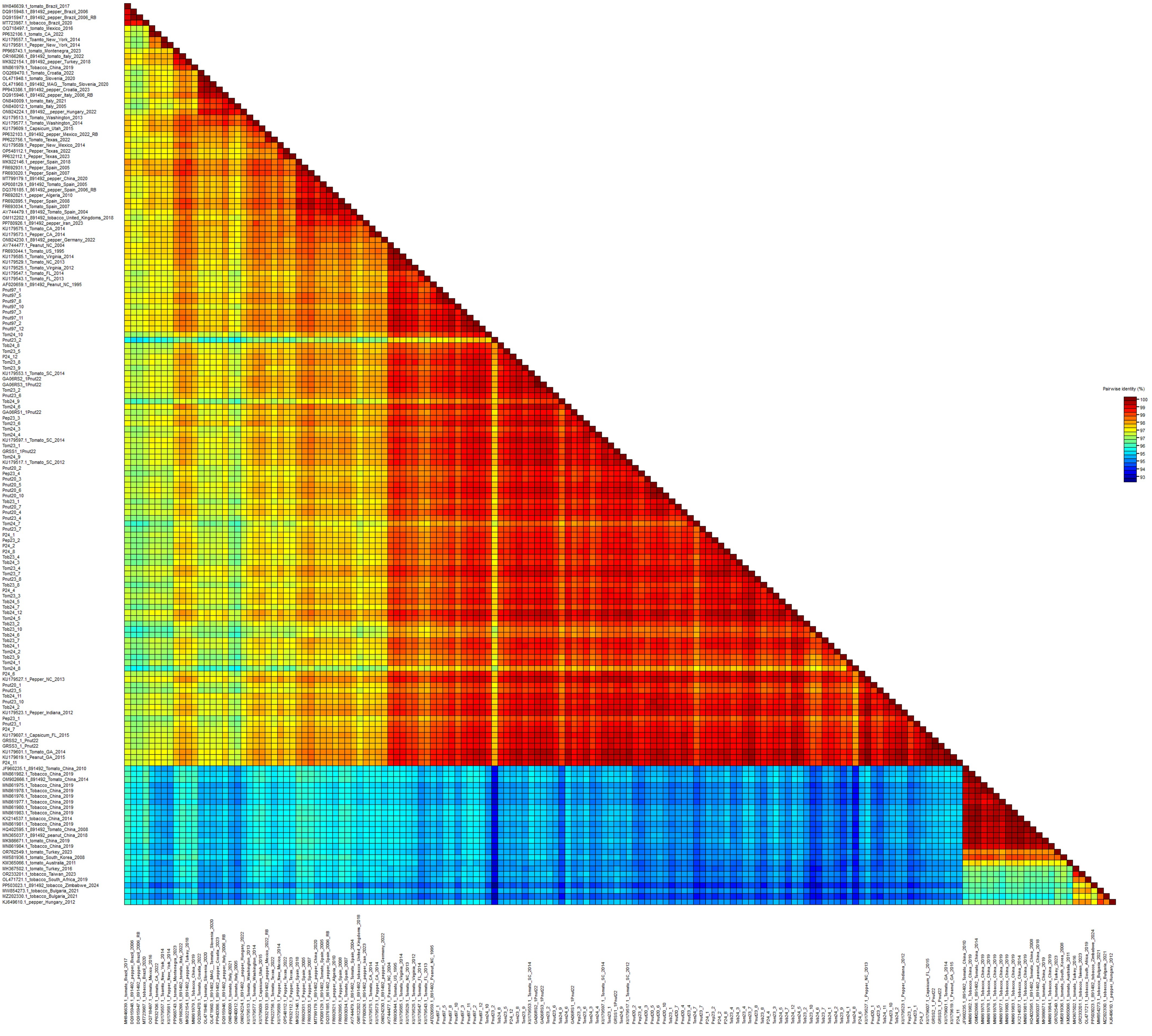

Fig S3a

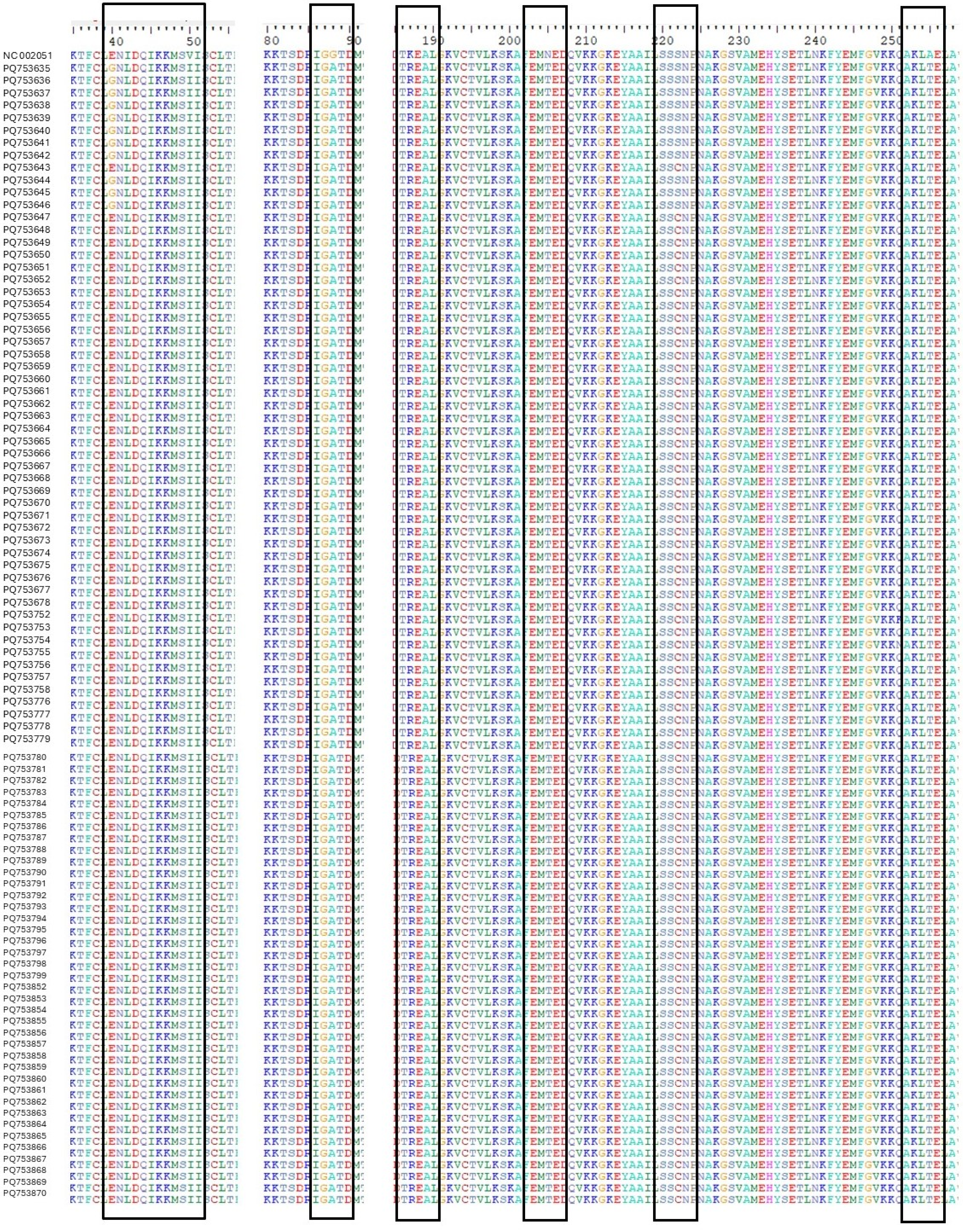

Fig S3b

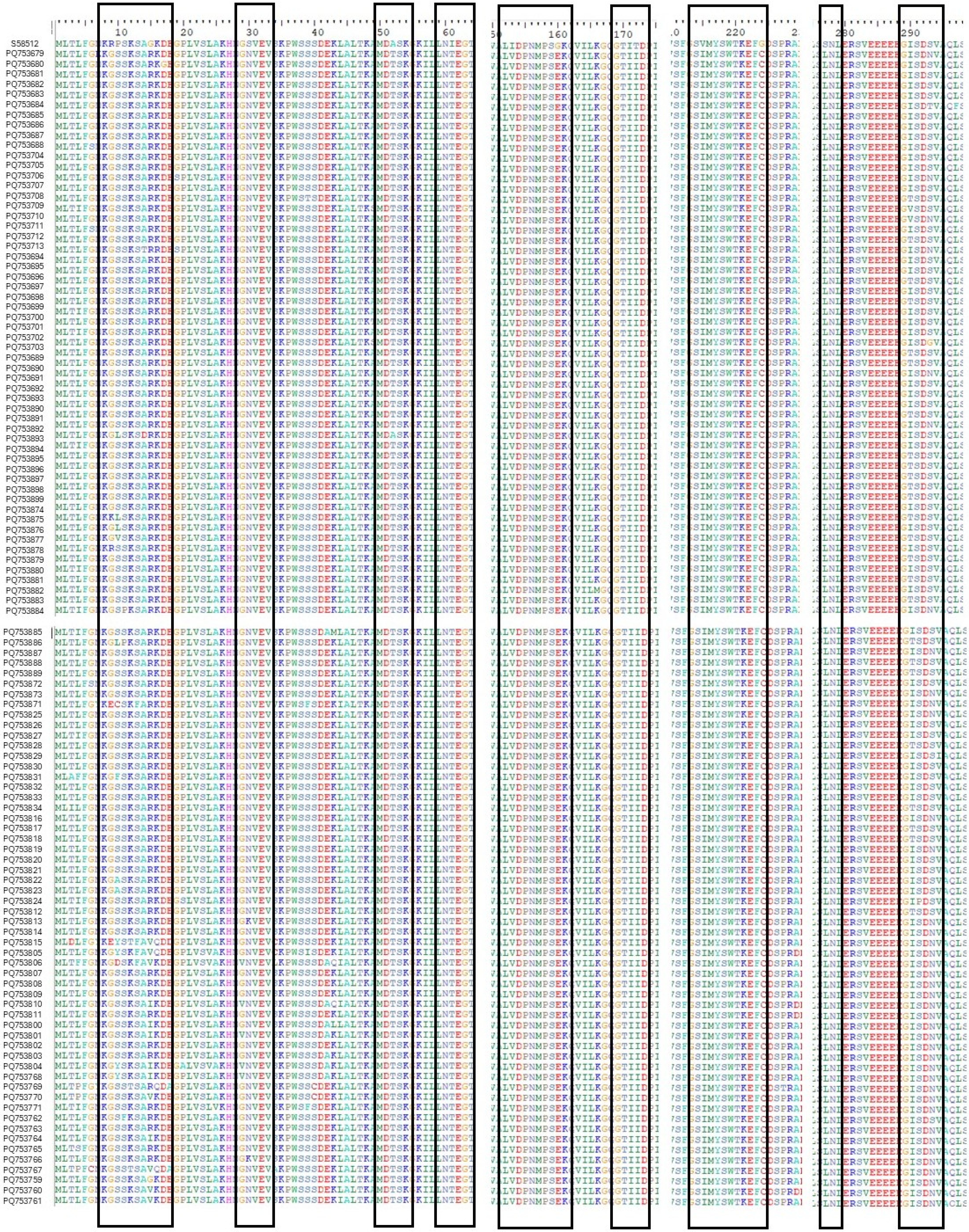

Fig S3c

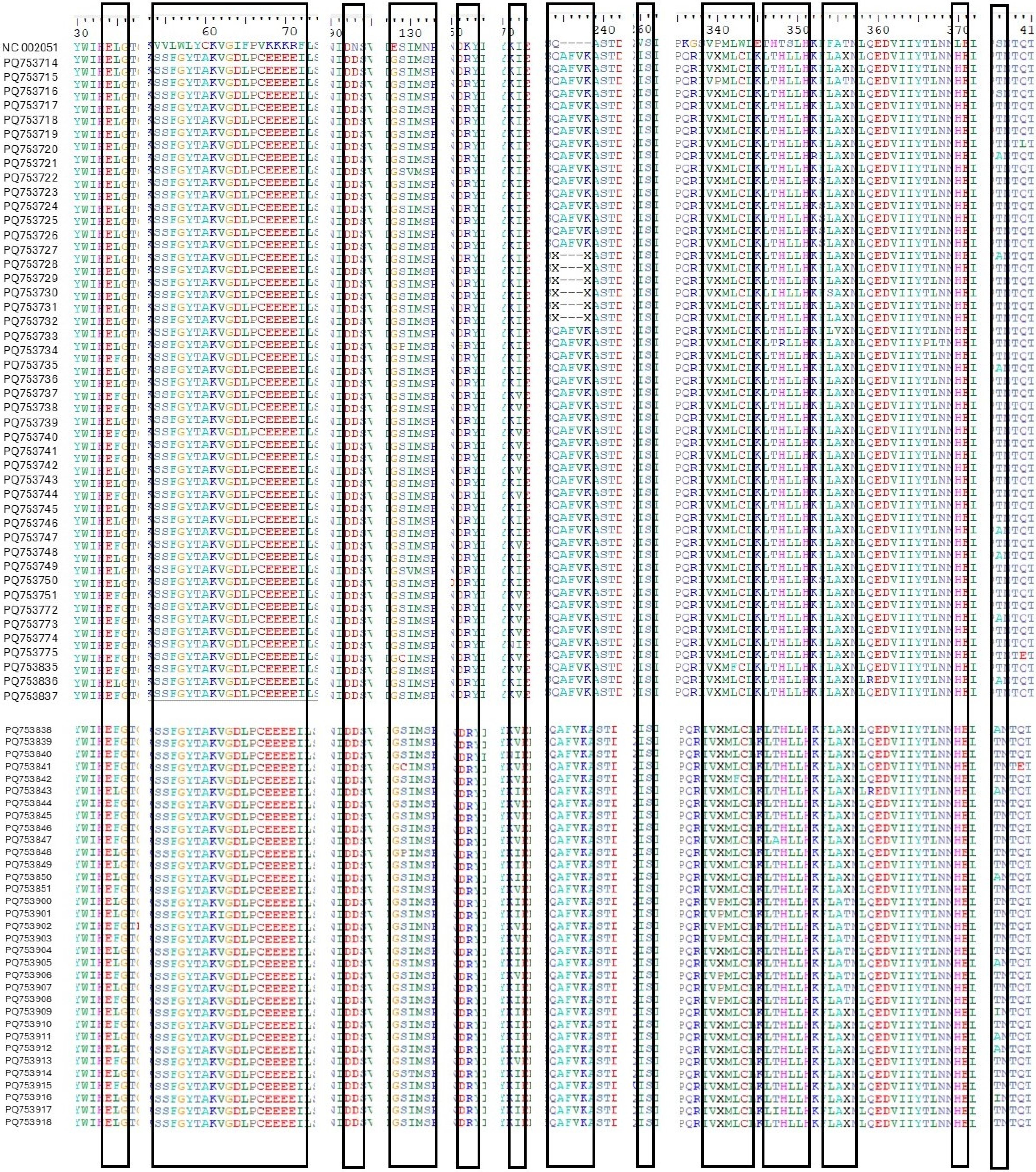
30
60
70
130
240
340
350
360
NC 002051
PQ753714
PQ753715
PQ753716
PQ753717
PQ753718
PQ753719
PQ753720
PQ753721
PQ753722
PQ753723
PQ753724
PQ753725
PQ753726
PQ753727
PQ753728
PQ753729
PQ753730
PQ753731
PQ753732
PQ753733
PQ753734
PQ753735
PQ753736
PQ753737
PQ753738
PQ753739
PQ753740
PQ753741
PQ753742
PQ753743
PQ753744
PQ753745
PQ753746
PQ753747
PQ753748
PQ753749
PQ753750
PQ753751
PQ753772
PQ753773
PQ753774
PQ753775
PQ753835
PQ753836
PQ753837
PQ753838
PQ753839
PQ753840
PQ753841
PQ753842
PQ753843
PQ753844
PQ753845
PQ753846
PQ753847
PQ753848
PQ753849
PQ753850
PQ753851
PQ753900
PQ753901
PQ753902
PQ753903
PQ753904
PQ753905
PQ753906
PQ753907
PQ753908
PQ753909
PQ753910
PQ753911
PQ753912
PQ753913
PQ753914
PQ753915
PQ753916
PQ753917
PQ753918

Fig S4a

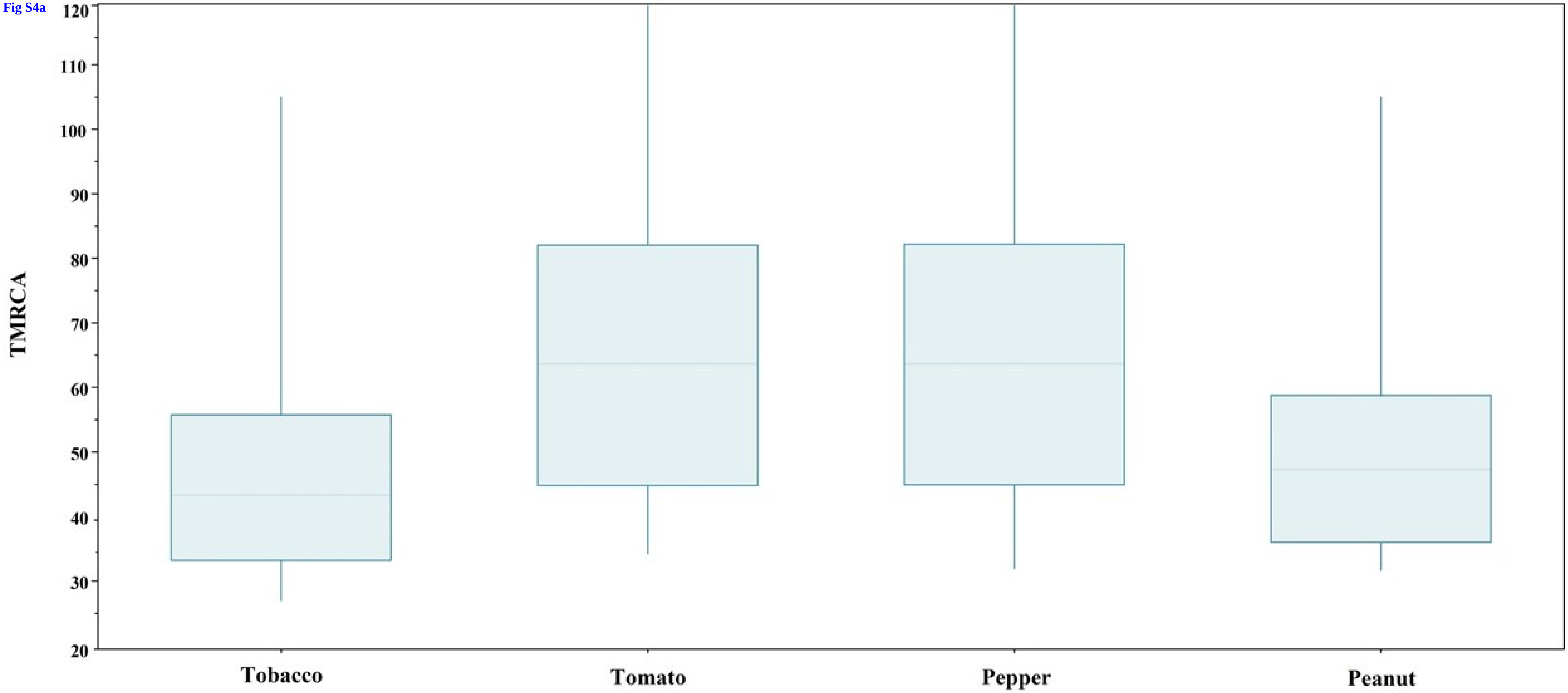

TMRCA
120
110
100
90
80
70
60
50
40
30
20
Tobacco
Tomato
Pepper
Peanut

Fig S4b

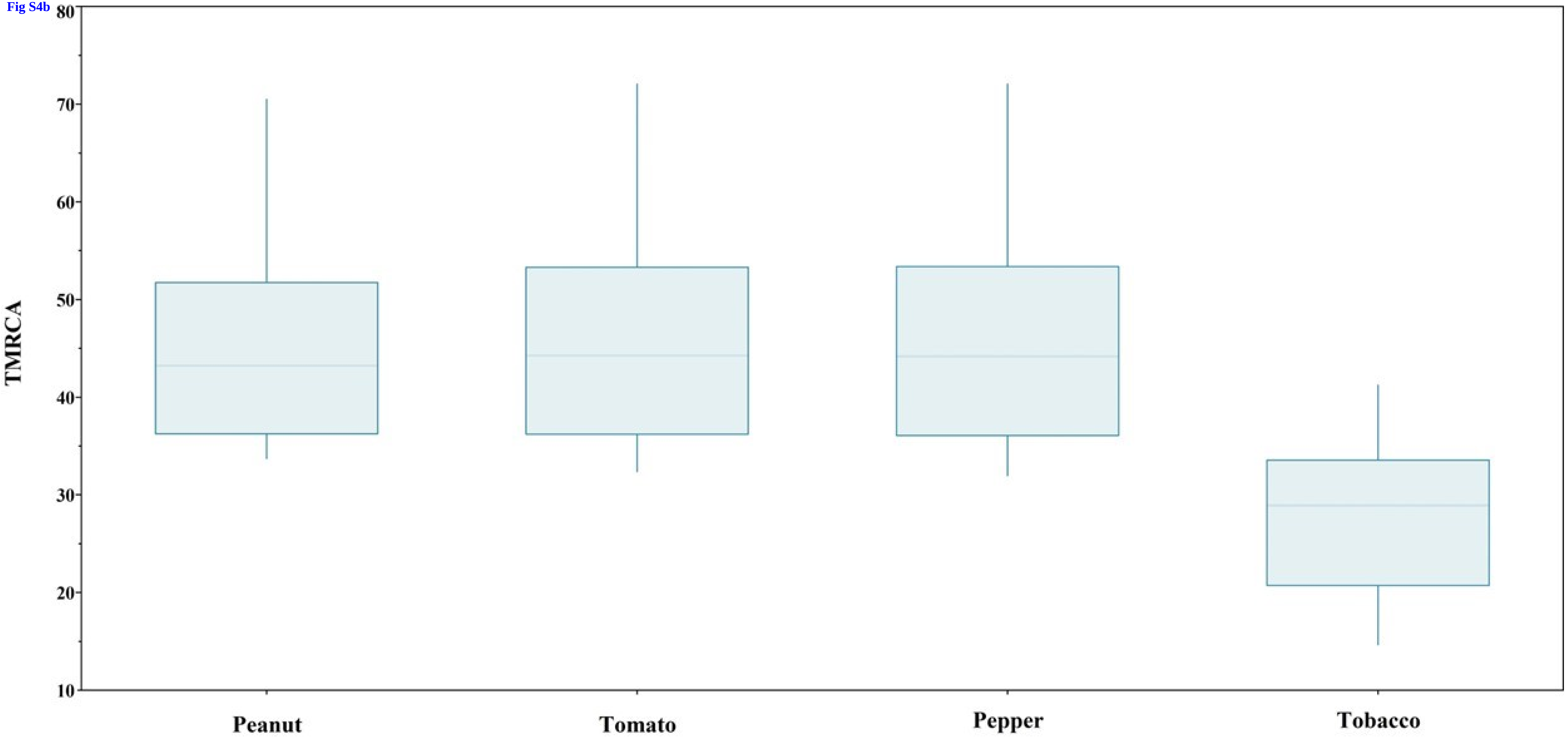

Fig S5a

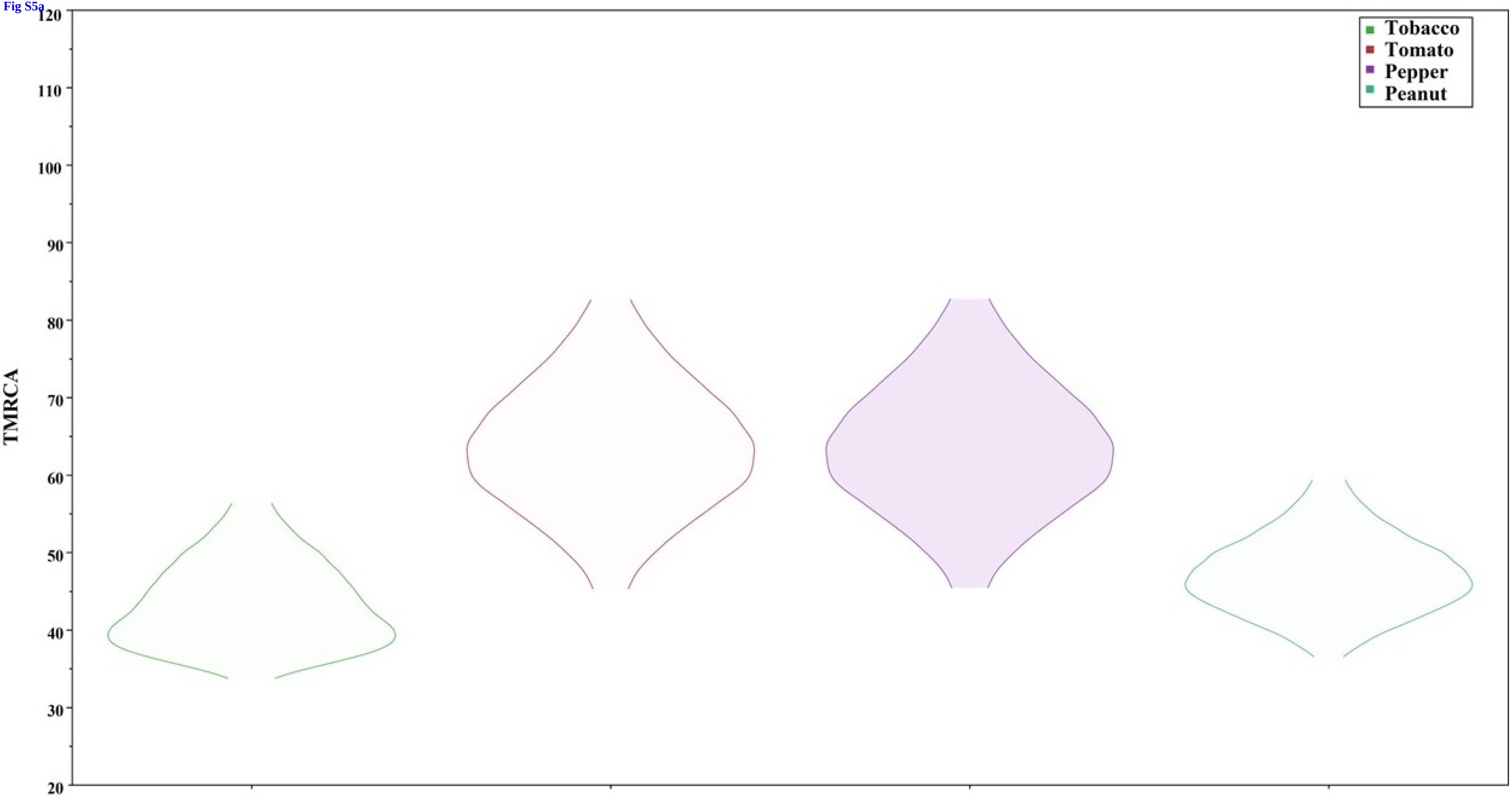

120
110
100
90
80
70
60
50
40
30
20
TMRCA
Tobacco
Tomato
Pepper
Peanut

Fig S5b

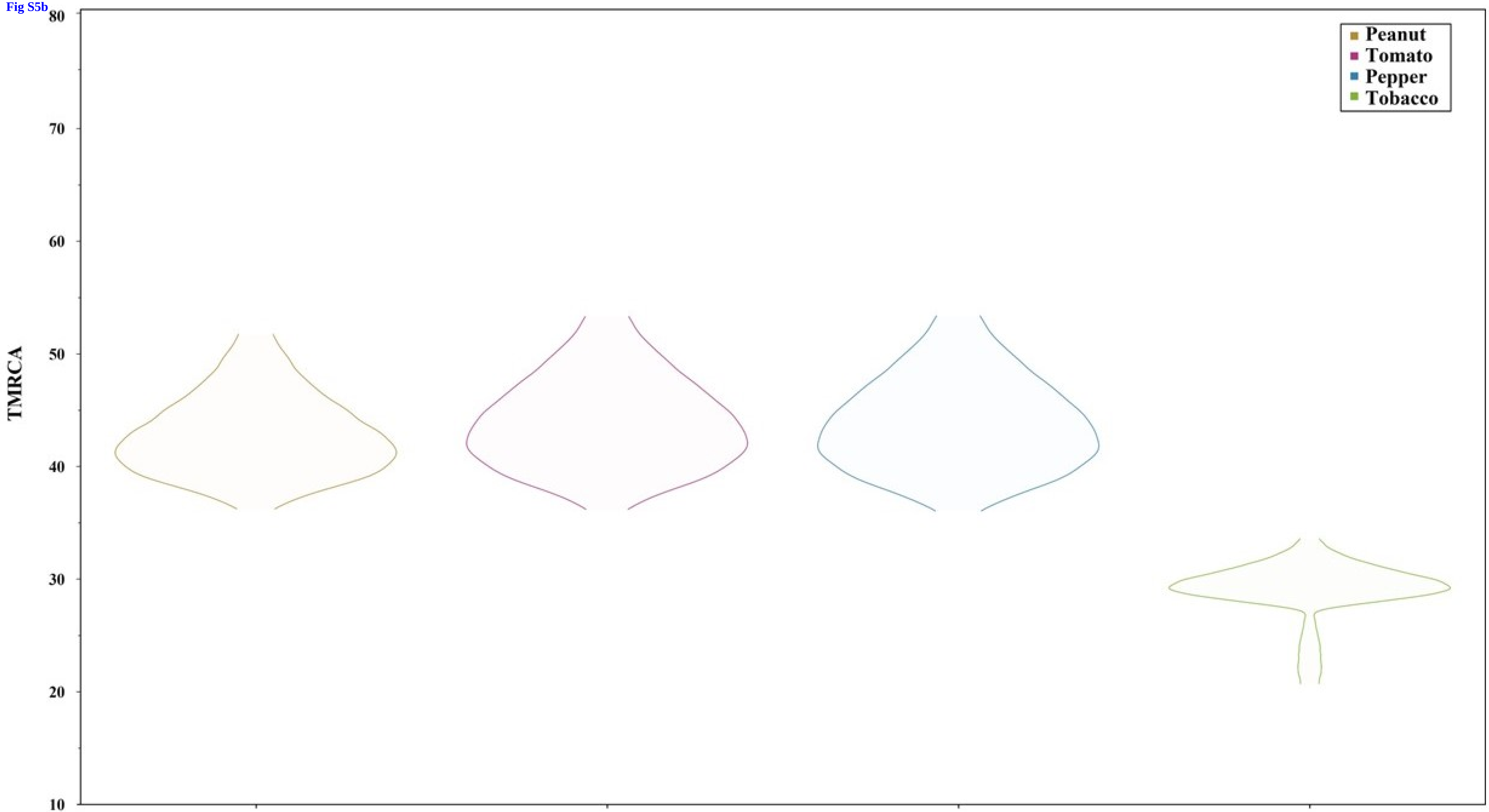

Fig S6a

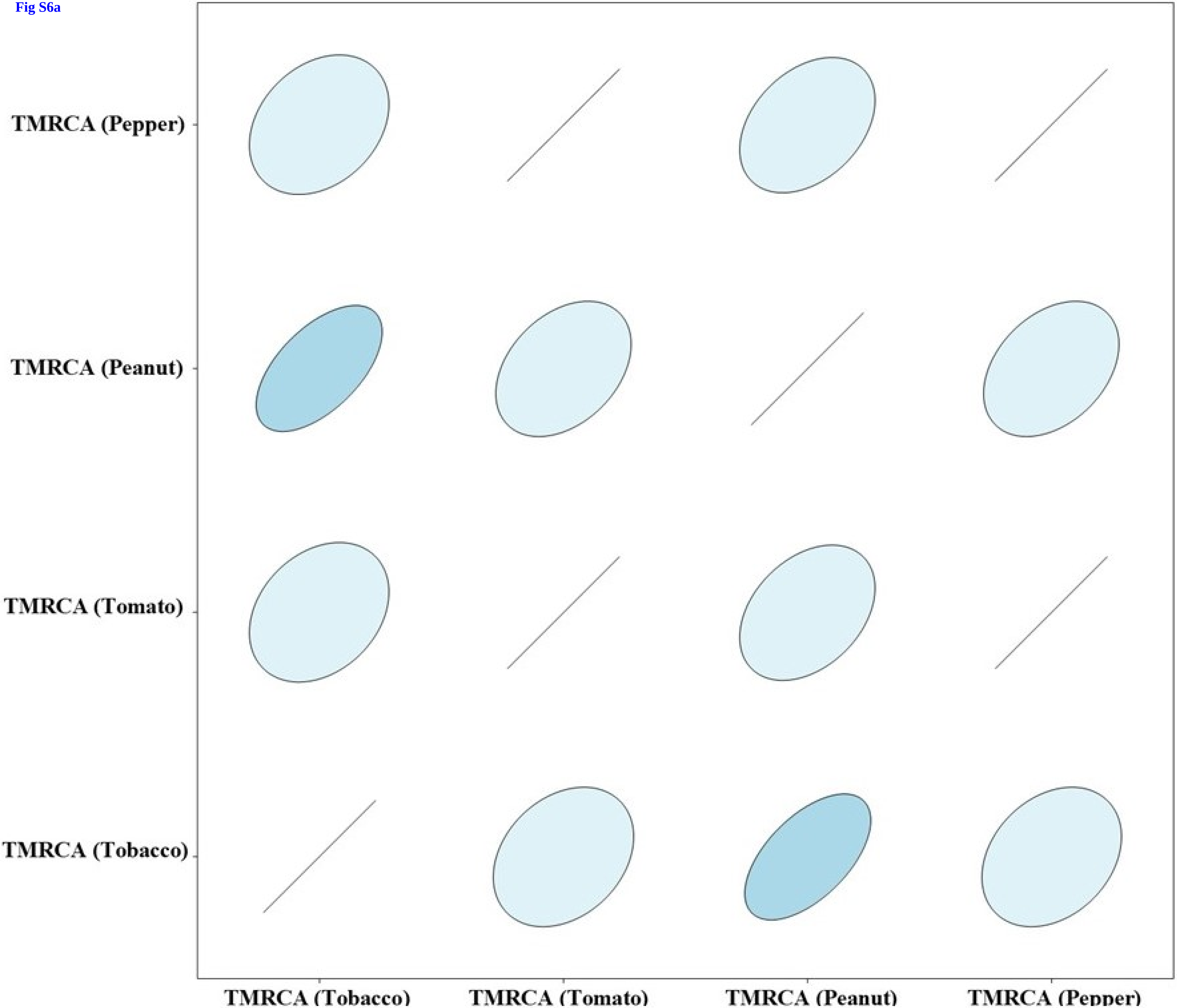

TMRCA (Pepper)
TMRCA (Peanut)
TMRCA (Tomato)
TMRCA (Tobacco)
TMRCA (Tobacco)
TMRCA (Tomato)
TMRCA (Peanut)
TMRCA (Pepper)

Fig S6b

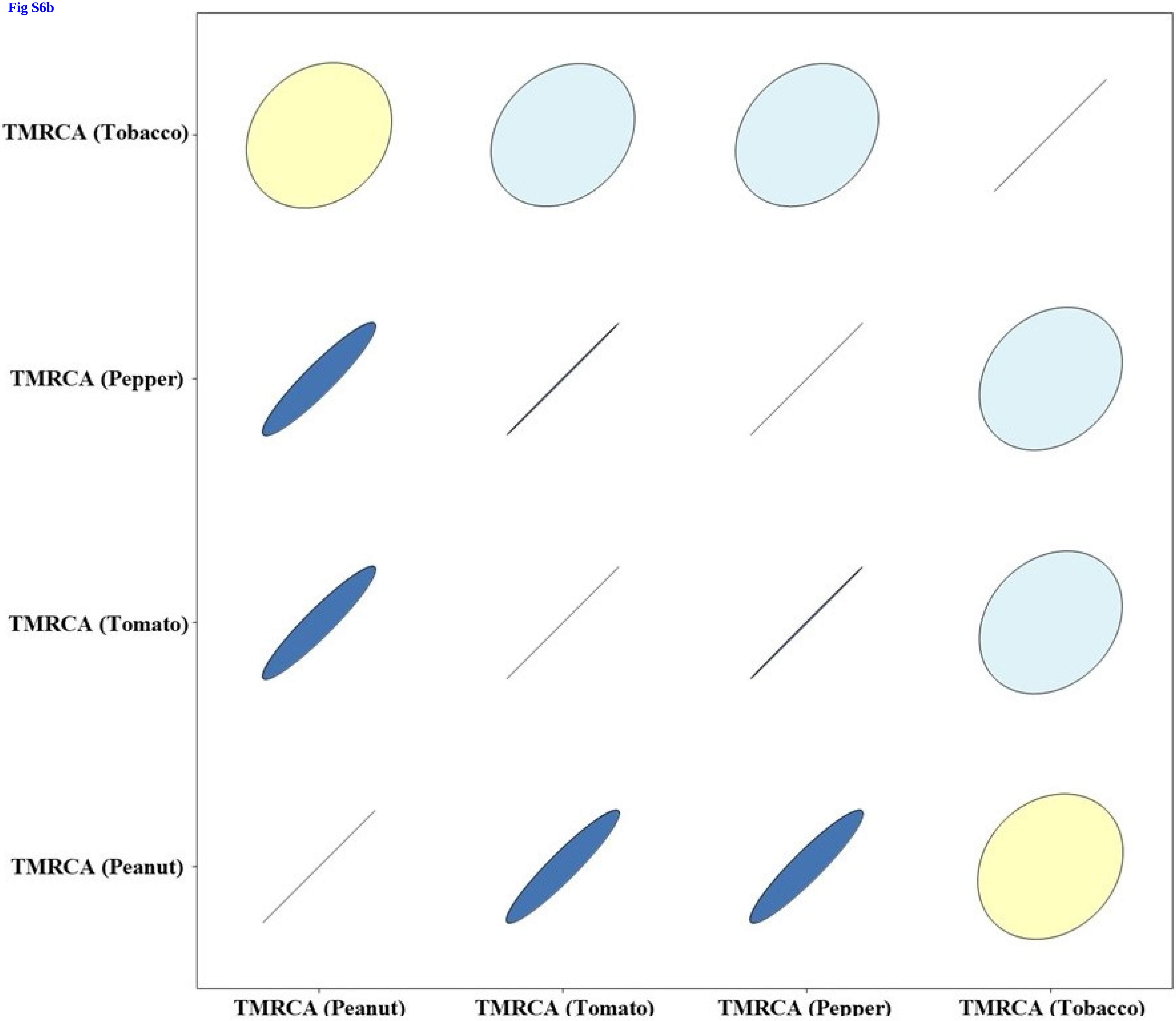
TMRCA (Tobacco)
TMRCA (Pepper)
TMRCA (Tomato)
TMRCA (Peanut)
TMRCA (Peanut)
TMRCA (Tomato)
TMRCA (Pepper)
TMRCA (Tobacco)

Supplementary data

Table Legends

Table S1. List of the primers used for amplification of full-length nucleocapsid (N), nonstructural movement (NSm), and nonstructural silencing suppressor (NSs) genes of tomato spotted wilt virus (TSWV).

Table S2. List of all the accession numbers (NCBI) of the sequences produced and used in this study.

Table S3. List of codons under positive selection in nucleocapsid (N), nonstructural movement (NSm), and nonstructural silencing suppressor (NSs) genes of tomato spotted wilt virus (TSWV).

Table S4. List of changes in amino acid residues observed in TSWV-GA isolates with differences in global representative sequences at same position in NSm and NSs genes of tomato spotted wilt virus, with respect to reference sequences.

Table S5. Summary statistics of time to the most recent common ancestor (TMRCA) of TSWV-US isolates based on **(a)** N (nucleocapsid protein) **(b)** NSm (non-structural movement protein), and **(c)** NSs (non-structural silencing-suppressor protein) and **(d)** N gene of TSWV-GA isolates using BEAST tool.

Table S1.

Primer name	Sequence (5'-3')	Amplicon size (bp)	Tm (ºC)
NSs_1_FP<br>NSs_1_RP	GTTCCTAATCAAACCTCACTTAG<br>GAGAGTTAGGCATGATGTTG	768	55
NSs_2_FP<br>NSs_2_RP	CCTAACAGAAATGTCCATGAATG<br>CAGGAAGGGATTTGGATGCTG	700	59
NSs_3_FP<br>NSs_3_RP	GGAGCCTTACAACATTGTTCATG<br>GCTTGTGCTTGCTTAGTGTG	901	58
N_FP<br>N_RP	CACACTAAGCAAGCACAAGC<br>GCACCTTGAATTACATACGGTC	909	59
NSm_FP<br>NSm_RP	AGAGCAATCAGTGCATCAGAA<br>GAGTCTTCGGACCAAAGT	1162	58

Table S2.

Crop	Gene	accession	location	year	Reference
Peanut	N	PQ753635 - PQ753646	GA	1997	This study
		PQ753647 - PQ753653	GA	2020	This study
		PQ753654 - PQ753659	GA	2022	This study
		PQ753660 - PQ753668	GA	2023	This study
		PQ753669 - PQ753678	GA	2024	This study
		AF048715	GA	1998	(Jain, Pappu et al. 1998)
		MW519215	GA	2018	NCBI
		HQ406903, HQ406934, HQ406956, HQ406958, HQ406966, HQ406970 - HQ406979, HQ406982, HQ406981	GA	2010	(Sundaraj, Srinivasan et al. 2014)
		OR364953, OR352894, OR349750	AL	2022	NCBI
		AY744477	NC	2004	(Tsompana, Abad et al. 2005)
		MN365037	China	2018	NCBI
		MK372884, MK372883	South Korea	2017-18	(Cho, Kim et al. 2020)
	NSm	PQ753679 - PQ753688	GA	1997	This study
		PQ753689 - PQ753693	GA	2020	This study

		PQ753694 - PQ753703	GA	2023	This study
		PQ753704 - PQ753713	GA	2024	This study
		KU179616, KU179618	GA	2015	NCBI
		MN365035	China	2018	NCBI
	NSs	PQ753714 - PQ753721	GA	1997	This study
		PQ753722 - PQ753729	GA	2020	This study
		PQ753730 - PQ753735	GA	2022	This study
		PQ753736 - PQ753743	GA	2023	This study
		PQ753744 - PQ753751	GA	2024	This study
		AF020659	NC	1995	(Qiu, Geske et al. 1998)
		AY744477	NC	2004	(Tsompana, Abad et al. 2005)
		KU179619	GA	2015	NCBI
Pepper	N	PQ753752 - PQ753754	GA	2020	This study
		PQ753755 - PQ753758	GA	2023	This study
		KU179527	NC	2013	NCBI
		KU179591	NM	2014	NCBI
		OR250147	TX	2023	NCBI
		FR693045	Algeria	2010	(Tentchev, Verdin et al. 2011)
		FR693121, FR693163	Spain	2007	(Tentchev, Verdin et al. 2011)
		AY818321	Australia	2002	NCBI
		MW293976	South Korea	2020	(Kwon, Cho et al. 2021)
		OP595391	South Korea	2022	NCBI
		ON924224	Hungary	2022	NCBI
		ON924230	Germany	2022	NCBI
		PP780926	Iran	2023	NCBI
		KM657114	China	2013	(Zhang, Wang et al. 2016)
		KT717693	Australia	2014	NCBI
		PP943380	Croatia	2023	NCBI
	NSm	PQ753759 - PQ753761	GA	2019	This study
		PQ753762 - PQ753767	GA	2020	This study

	PQ753768 - PQ753771	GA	2023	This study
	PP632108	TX	2022	NCBI
	PP632111	TX	2023	NCBI
	KU179612	FL	2015	NCBI
	KU179608	UT	2015	NCBI
	KU179588	NM	2014	NCBI
	KU179580	NY	2014	NCBI
	KU179566	CA	2014	NCBI
	KU179522	IN	2012	NCBI
	MT723986	Brazil	2020	NCBI
	MZ404051	South Korea	2020	NCBI
	OM112201	United Kingdom	2018	(Pecman, Kutnjak et al. 2017)
	KT717692	Australia	2014	NCBI
	KM657117	China	2013	NCBI
	MG025803	Australia	2015	NCBI
	PP943385	Croatia	2023	NCBI
	PP780925	Iran	2023	NCBI
	OM867574	Italy	2021	NCBI
	MH756624	Italy	2014	NCBI
	MF326510	South Korea	2016	NCBI
	HM581941	South Korea	2009	(Lee, Cho et al. 2011)
	AB650468	South Korea	2011	NCBI
**NSs**	PQ753772 - PQ753775	GA	2023	This study
	KU179527	NC	2013	NCBI
	KU179523	IN	2012	NCBI
	KU179573	CA	2014	NCBI
	KU179581	NY	2014	NCBI
	KU179589	NM	2014	NCBI
	KU179609	UT	2015	NCBI
	KU179607	FL	2015	NCBI
	OP548112	TX	2022	(Gautam, Chinnaiah et al. 2023)
	MK922146	Spain	2018	NCBI

		FR693020	Spain	2007	(Tentchev, Verdin et al. 2011)
		FR692931	Spain	2005	(Tentchev, Verdin et al. 2011)
		FR692895	Spain	2008	(Tentchev, Verdin et al. 2011)
		MK922154	Turkey	2018	NCBI
		DQ376185	Spain	2006	NCBI
		MT799179	China	2020	NCBI
		ON924230	Germany	2022	NCBI
		PP632103	Mexico	2022	NCBI
		DQ915947, DQ915948	Brazil	2006	NCBI
		DQ915946	Italy	2006	NCBI
		PP943386	Croatia	2023	NCBI
		KJ649610	Hungary	2012	(Almási, Csilléry et al. 2015)
		ON924224	Hungary	2022	
		FR692821	Algeria	2010	(Tentchev, Verdin et al. 2011)
		PP780926	Iran	2023	NCBI
**Tomato**	**N**	PQ753852 - PQ753860	GA	2023	This study
		PQ753861 - PQ753870	GA	2024	This study
		AF048714	GA	1998	(Jain, Pappu et al. 1998)
		KU179601	GA	2014	NCBI
		HQ406983	GA	2010	(Sundaraj, Srinivasan et al. 2014)
		OR250143	TX	2022	NCBI
		PP632106	CA	2022	NCBI
		KU179513	WA	2013	NCBI
		KU179531	NC	2013	NCBI
		KU179517	SC	2012	NCBI
		PP968743	Montenegro	2023	NCBI

	KM365066	Australia	2011	(Wylie, Zhang et al. 2015)
	FR693265	Spain	2007	(Tentchev, Verdin et al. 2011)
	OQ718497	Mexico	2016	(Rodríguez-Negrete, Guevara-Rivera et al. 2023)
	MZ568850	Turkey	2020	(Morca, Çelik et al. 2022)
	MN938463	China	2020	NCBI
	MT723987	Brazil	2020	NCBI
	MN966569	Pakistan	2018	NCBI
	KP008129	Spain	2005	(Debreczeni, López et al. 2015)
	OR166266	Italy	2022	NCBI
	OL471948	Slovenia	2020	NCBI
	ON840009	Italy	2021	NCBI
	JF960235	China	2010	(Hu, Feng et al. 2011)
	MK986671	China	2019	NCBI
	OQ269470	Croatia	2022	NCBI
	D00645	Brazil	1989	(De Haan, Wagemakers et al. 1989)
	PQ753871	GA	2019	This study
	PQ753872, PQ753873	GA	2020	This study
	PQ753874 - PQ753889	GA	2023	This study
	PQ753890 - PQ753899	GA	2024	This study
	PP632105	CA	2024	NCBI
**NSm**	PP622752	TX	2022	
	PP105569, PP105571, PP105572, PP105574, PP105575	CA	2023	(Macedo, Melgarejo et al. 2024)
	PP105567	NJ	2022	(Macedo, Melgarejo et al. 2024)

	PP105562	CA	2014	(Macedo, Melgarejo et al. 2024)
	OP832373	NC	2022	(Lahre, Shekasteband et al. 2023)
	KX898454	CA	2016	(Batuman, Turini et al. 2017)
	KU179592	SC	2014	NCBI
	KU179560, KU179574	CA	2014	NCBI
	KU179556	NY	2014	NCBI
	KU179550	FL	2014	NCBI
	KU179544	FL	2013	NCBI
	KU179542	FL	2013	NCBI
	KU179516	SC	2012	NCBI
	AY956380	FL	2005	(Lewandowski and Adkins 2005)
	PP632102	Mexico	2022	NCBI
	PP540042	Italy	2011	NCBI
	PP105564	Mexico	2014	(Macedo, Melgarejo et al. 2024)
	OQ718487	Mexico	2016	(Rodríguez-Negrete, Guevara-Rivera et al. 2023)
	OQ507122	Croatia	2022	NCBI
	OP373185	Croatia	2021	NCBI
	ON840011	Italy	2005	NCBI
	OM902667	China	2014	NCBI
	MK887284	China	2019	NCBI
	MK792775	France	2005	(Terret-Welter, Bonnet et al. 2020)
	KM365065	Australia	2011	(Wylie, Zhang et al. 2015)

	JF960236	China	2010	(Hu, Feng et al. 2011)
	HM015516	Australia	2010	(Lopez, Aramburu et al. 2011)
	FM163373	Spain	2005	(Lopez, Aramburu et al. 2011)
	AY744492, AY744493	Spain	2004	(Tsompana, Abad et al. 2005)
**NSs**	PQ753900 - PQ753908	GA	2023	This study
	PQ753909 - PQ753918	GA	2024	This study
	FR693044	US	1995	(Tentchev, Verdin et al. 2011)
	KU179517	SC	2012	NCBI
	KU179513	WA	2013	NCBI
	KU179547	FL	2014	NCBI
	KU179543	FL	2013	NCBI
	KU179529	NC	2013	NCBI
	KU179525	VA	2012	NCBI
	KU179553	SC	2014	NCBI
	KU179557	NY	2014	NCBI
	KU179577	WA	2014	NCBI
	KU179575	CA	2014	NCBI
	KU179585	VA	2014	NCBI
	KU179597	SC	2014	NCBI
	KU179601	GA	2014	NCBI
	PP632106	CA	2022	NCBI
	PP622756	TX	2022	NCBI
	KP008129	Spain	2005	(Debreczeni, López et al. 2015)
	OR166266	Italy	2022	NCBI
	FR693034	Spain	2007	(Tentchev, Verdin et al. 2011)

		AY744479	Spain	2004	(Tsompana, Abad et al. 2005)
		OM902666	China	2014	NCBI
		JF960235	China	2010	(Hu, Feng et al. 2011)
		HQ402595	China	2008	NCBI
		MK986671	China	2019	NCBI
		HM581936	South Korea	2008	(Lee, Cho et al. 2011)
		KM365066	Australia	2011	(Wylie, Zhang et al. 2015)
		OQ718497	Mexico	2016	(Rodríguez-Negrete, Guevara-Rivera et al. 2023)
		PP968743	Montenegro	2023	NCBI
		MH367502	Turkey	2016	(Fidan and Sari 2019)
		OR762549	Turkey	2023	NCBI
		OQ269470	Croatia	2022	NCBI
		OP373184	Croatia	2021	NCBI
		MH846636, MH846639	Brazil	2017	NCBI
		ON840012	Italy	2005	NCBI
		ON840009	Italy	2021	NCBI
		OL471948, OL471960	Slovenia	2020	NCBI
**Tobacco**	**N**	PQ753776, PQ753777	GA	2019	This study
		PQ753778, PQ753779	GA	2020	This study
		PQ753780 - PQ753789	GA	2023	This study
		PQ753790 - PQ753799	GA	2024	This study
		AF064470	GA	1998	(Pappu, Pappu et al. 1998)
		HQ406984	GA	2010	(Sundaraj, Srinivasan et al. 2014)
		HQ406904	NC	2010	(Sundaraj, Srinivasan et al. 2014)
		OL471719	South Africa	2019	NCBI

	MW854273	Bulgaria	2021	NCBI
	OR233201	Taiwan	2023	NCBI
	MT723987	Brazil	2020	NCBI
	MZ404052	South Korea	2020	NCBI
	PP503023	Zimbabwe	2024	NCBI
	OM112202	United Kingdom	2018	(Pecman, Kutnjak et al. 2017)
	MT723987	Brazil	2020	NCBI
	MN861975 - MN861984	China	2019	(Kamran, Li et al. 2024)
	KM657115	China	2013	NCBI
**NSm**	PQ753800 - PQ753804	GA	2018	This study
	PQ753805 - PQ753811	GA	2019	This study
	PQ753812 - PQ753815	GA	2020	This study
	PQ753816 - PQ753824	GA	2023	This study
	PQ753825 - PQ753834	GA	2024	This study
	AY744487 - AY744490	NC	2004	(Tsompana, Abad et al. 2005)
	MN870627 - MN870632, MN870637, MN870639, MN833253	China	2019	(Kamran, Li et al. 2024)
	ON496935	China	2019	NCBI
	PP503022	Zimbabwe	2024	NCBI
	MZ202329, MW854272	Bulgaria	2021	NCBI
**NSs**	PQ753835 - PQ753841	Georgia	2023	This study
	PQ753842 - PQ753851	Georgia	2024	This study
	OL471719, OL471721	South Africa	2019	NCBI
	MZ202330, MW854273	Bulgaria	2021	NCBI
	MT723987	Brazil	2020	NCBI
	OR233201	Taiwan	2023	NCBI
	MN861975 - MN861984	China	2019	(Kamran, Li et al. 2024)
	KX214537	China	2014	NCBI
	PP503023	Zimbabwe	2024	NCBI
	OM112202	United Kingdom	2018	(Pecman, Kutnjak et al. 2017)

Table S3.

**Gene**	**Isolates**	**Codons**	**dN/dS**	***p*-value**	**Position**
**N**	TSWV-GA (peanut, pepper, tobacco, and tomato)	6th	8.36	0.198	L6S
**NSm**	TSWV-US (Pepper)	15th	16.8	0.155	R15V, G, I
		4th	16.6	0.142	L4I, F, P, S
	TSWV-US (Tobacco)	10th	16.4	0.097	S10L, C, V
	TSWV-US (Tomato)	10th	10.7	0.144	S10L, C, V
		9th	7.15	0.198	G9K, E
	TSWV-GA (peanut, pepper, tobacco, and tomato)	15th	3.4	0.091	R15I, V, G
		10th	9.57	0.111	S10Y, L, C, V, D, A, F
		43rd	5.22	0.196	K43Q, L, M
		3rd	4.32	0.199	T3I, D, A
		6th	4.32	0.199	G6S, C
**NSs**	TSWV-US (Pepper)	405th	21.8	0.198	S405T, A
	TSWV-GA (peanut, pepper, tobacco, and tomato)	437th	10.1	0.065	S437F, C
		434th	7.28	0.134	S434F, P
		463rd	7.27	0.185	Y434H
		406th	7.29	0.197	T406A, I, S

Note: standard one letter abbreviations are used for amino acids in this table.

Table S4

Sr. No.	Associated functions/domains	Codon position	Ref. Seq. NSs (NC002051)	Amino acid changes			Amino acid changes		
				Local population	Accession no.	Location/Crop/Year	Global population	Accession no.	Location/Crop/Year
1	Predicted RNA binding domain (mutation caused loss of HR)	48th	S (Polar; uncharged)	F (Non-polar; uncharged)	PQ753772 and PQ753775	Georgia; Pepper; 2023	S	Conserved in all sequences	Peanut, Pepper, Tobacco and Tomato
2	Predicted RNA binding domain (mutation caused loss of HR and partial activity of RSS)	197th/198th	Y/S (Polar; uncharged)	S/S	PQ753911	Georgia; Tomato; 2024	Y/C (Polar; uncharged)	HM581936	South Korea; Tomato; 2008
				Y/G (Polar; uncharged)	PQ753740	Georgia; Peanut; 2023		HQ402595	China; Tomato; 2008
								JF960235	China; Tomato; 2010
								OM902666	China; Tomato; 2014
								KX214537	China; Tobacco; 2014
								MN365037	China; Peanut; 2018
								MN861975-MN861984	China; Tobacco; 2019
								MK986671	China; Tomato; 2019
								OR762549	Turkey; Tomato; 2023
3	Conserved NSs domain (mutation caused loss of HR and RSS)	368th/369th	N/N (Polar; uncharged)	T/N (Polar; uncharged)	PQ753737		N/N	Conserved in all sequences	
4	Predicted RNA binding domain (mutation caused loss of HR)	411th/412th	S/Y (Polar; uncharged)	C/Y (Polar; uncharged)	PQ753916		S/Y	Conserved in all sequences	
				F/Y (Polar; uncharged)	PQ753846, PQ753836 and PQ753740	Georgia; Tobacco; 2024 and Georgia; Peanut; 2023			

Cont.

5	N-terminal	35th	L (non-polar; uncharged)	F (non-polar; uncharged)	PQ753738, PQ753741, PQ753742	Georgia; Peanut; 2023	L	Conserved in all sequences
					PQ753744-PQ753746, and PQ753749	Georgia; Peanut; 2024		
					PQ753773	Georgia; Pepper; 2023		
					PQ753836-PQ753841	Georgia; Tobacco; 2023		
					PQ753842, PQ753844-PQ753847 and PQ753851	Georgia; Tobacco; 2024		
					PQ753902, PQ753903, PQ753906 and PQ753908	Georgia; Tomato; 2023		
					PQ753910, PQ753913, and PQ753915	Georgia; Tomato; 2024		

Cont.

6	N-terminal	132nd	N (Polar; uncharged)	S (Polar; uncharged)	Present in all the selected sequences except PQ753902	Georgia; Tomato; 2023	S (Polar; uncharged)	FR693044	US; Tomato; 1995
								AF020659	NC; Peanut; 1995
								AY744477	NC; Peanut; 2004
								KU179517	SC; Tomato; 2012
								KU179523	IN; Pepper; 2012
								KU179525	VA; Tomato; 2012
								KU179527	NC; Pepper; 2013
								KU179529	NC; Tomato; 2013
								KU179543	FL; Tomato; 2013
								KU179585	VA; Tomato; 2014
								KU179597	SC; Tomato; 2014
								KU179601	GA; Tomato; 2014
								KU179547	FL; Tomato; 2014
								KU179619	GA; Peanut; 2015
								KU179607	FL; Pepper; 2015

Cont.

Sr. No.	Associated functions/domains	Codon position	Reference Seq NSm (S58512)	Amino acid changes			Amino acid changes		
				Local population	Accession no.	Location/Crop/Year	Global population	Accession no.	Location/Crop/ Year
1	Tubule formation and cell-to-cell movement related region	G19 - S159							
		19th	G (Polar; uncharged)	S (Polar; uncharged)	PQ753671, PQ753678	Georgia; Peanut; 2024			
		20th	P (non-polar; uncharged)	A (non-polar; uncharged)	PQ753803	Georgia; Tobacco; 2018			
				S (Polar; uncharged)	PQ753823	Georgia; Tobacco; 2023			
		29th	G (Polar; uncharged)	V (non-polar; uncharged)	PQ753804, PQ753803, PQ753800, PQ753809, PQ753805	Georgia; Tobacco; 2018 and Georgia; Tobacco; 2019			
		30th	S (Polar; uncharged)	K (Basic; Charged)	PQ753812, PQ753765	Georgia; Tobacco; 2018 and Georgia; Pepper; 2020			
				N (Polar; uncharged)	PQ753635-PQ753764, PQ753766-PQ753811, PQ753813-PQ753918	Georgia; Peanut; Pepper; Tobacco; Tomato; 1997, 2018-20, 2023-24			
		34th	S (Polar; uncharged)	A (non-polar; uncharged)	PQ753766	Georgia; Pepper; 2020			
				L (non-polar; uncharged)	PQ753805	Georgia; Tobacco; 2019			
				C (Polar; uncharged)	PQ753814, PQ753815	Georgia; Tobacco; 2020			

Cont.

1	Tubule formation and cell-to-cell movement related region	34th	S (Polar; uncharged)	A (non-polar; uncharged)	PQ753766	Georgia; Pepper; 2020			
				L (non-polar; uncharged)	PQ753805	Georgia; Tobacco; 2019			
				C (Polar; uncharged)	PQ753814 and PQ753815	Georgia; Tobacco; 2020			
		39th	S (Polar; uncharged)	F (non-polar; uncharged)	PQ753873 and PQ753770	Georgia; Tomato; 2020 and Georgia; Pepper; 2023			
				I (non-polar; uncharged)	PQ753765	Georgia; Tobacco; 2020			
				T (Polar; uncharged)	PQ753708	Georgia; Peanut; 2024			
		40th	S (Polar; uncharged)	C (Polar; uncharged)	PQ753768 and PQ753769	Georgia; Pepper; 2023	A (Non-polar; uncharged)	AY744493	Spain; Tomato; 2004
							F (Non-polar; uncharged)	MH756624	Italy; Pepper; 2014
		42nd	E (Acidic; Charged)	A (non-polar; uncharged)	PQ753800, PQ753802-PQ753805, PQ753809, PQ753811 and PQ753885	Georgia; Tobacco; 2018-19 and Georgia; Tomato; 2023			
		43rd	K (Basic; Charged)	Q (Polar; uncharged)	PQ753805 and PQ753809	Georgia; Tobacco; 2019			
				L (non-polar; uncharged)	PQ753811	Georgia; Tobacco; 2019			
				M (non-polar; uncharged)	PQ753885	Georgia; Tomato; 2023			

Cont.

1	Tubule formation and cell-to-cell movement related region	44th	L (non-polar; uncharged)	I (non-polar; uncharged)	PQ753760, PQ753805, PQ753809, PQ753763, PQ753766-PQ753767, PQ753814-PQ753815, and PQ753873	Georgia; Pepper; 2019-20, 2023, Georgia; Tobacco; 2019-20, and Georgia; Tomato; 2020			
		49th	A (non-polar; uncharged)	T (Polar; uncharged)	PQ753694, PQ753700	Georgia; Peanut; 2023	V (non-polar; uncharged)	KU179542 and KU179544	FL; Tomato; 2013
				S (Polar; uncharged)	PQ753702, PQ753709	Georgia; Peanut; 2023-24			
		52nd	A (non-polar; uncharged)	T (Polar; uncharged)	PQ753635-PQ753918	Georgia; Peanut; Pepper; Tobacco; Tomato; 1997, 2018-20, 2023-24			
2	Foliar necrosis	I57 - N100							
		75	I (non-polar; uncharged)	S (Polar; uncharged)	PQ753765, PQ753812	Georgia; Pepper; 2020, Georgia; Tobacco; 2020			
		110	N (Polar; uncharged)	T (Polar; uncharged)	PQ753816, PQ753682	Georgia; Tobacco; 2024, Georgia; Peanut; 1997	T (Polar; uncharged)	KU179608	UT; Pepper; 2015
		114	V (non-polar; uncharged)	I (non-polar; uncharged)	PQ753830, PQ753833, PQ753696, PQ753703, PQ753712, PQ753686	Georgia; Tobacco; 2024, Georgia; Peanut; 1997, 2023-24	I (Polar; uncharged)	PP105564	Mexico; Tomato; 2014
								OQ507122	Croatia; Tomato; 2022
3	Tubule formation and cell-to-cell movement related region	G209 - V284							
		215	V (non-polar; uncharged)	I (non-polar; uncharged)	PQ753635-PQ753918	Georgia; Peanut; Pepper; Tobacco; Tomato; 1997, 2018-20, 2023-24	I (Polar; uncharged)		
		229	A (non-polar; uncharged)	D (Acidic; Charged)	PQ753759, PQ753810, PQ753809, PQ753815	Georgia; Pepper; 2019, Georgia; Tobacco; 2020			
		234	S (Polar; uncharged)	N (Polar; uncharged)	PQ753696, PQ753703	Georgia; Peanut; 2023			
		277	S (Polar; uncharged)	L (non-polar; uncharged)	PQ753635-PQ753918	Georgia; Peanut; Pepper; Tobacco; Tomato; 1997, 2018-20, 2023-24	S (Polar; uncharged)		

Table S5.

Summary Statistic	(a) TMRCA estimates for TSWV-N gene			
	**Peanut**	**Pepper**	**Tobacco**	**Tomato**
Mean	46.5312	46.504	44.0822	46.1517
Stderr of mean	0.1318	0.1319	0.1479	0.1313
Std dev	5.5825	5.595	6.2954	5.6517
Variance	31.648	31.3036	39.6327	31.9418
Median	45.7327	45.7083	43.8813	45.3682
Geometric mean	46.2131	46.1843	43.6359	45.823
95% HPD interval	[36.7326, 57.67.48]	[36.7023, 57.6907]	[30.8812, 55.4136]	[36.2469, 57.4077]
Auto-correlation time (ACT)	37630.4499	37490.4925	37273.5142	36423.4093
Effective sample size (ESS)	1793.8	1800.5	1811	1853.2
Number of samples	67501	67501	67501	67501

Summary Statistic	(b) TMRCA estimates for TSWV-NSm gene			
	**Tobacco**	**Tomato**	**Pepper**	**Peanut**
Mean	43.878	64.2024	64.2025	47.8158
Stderr of mean	0.0888	0.2057	0.2058	0.1006
Std dev	6.5046	9.4206	9.4245	6.1766
Variance	42.3102	88.7472	88.8215	38.1509
Median	42.7661	63.6987	63.6987	47.11
Geometric mean	43.4303	63.518	63.5155	47.4426
95% HPD interval	[33.77, 56.3097]	[45.3493, 82.5737]	[45.481, 82.7203]	[36.5767, 59.3095]
Auto-correlation time (ACT)	12569.7263	32189.3086	32192.7676	17902.9939

Effective sample size (ESS)	5370.1	2097	2096.8	37770.4
Number of samples	67501	67501	67501	67501

Summary Statistic	(c) TMRCA estimates for TSWV-NSs gene			
	Peanut	Tomato	Pepper	Tobacco
Mean	43.2034	44.244	44.142	28.8958
Stderr of mean	0.0854	0.1182	0.1198	0.0355
Std dev	4.1726	4.599	4.6634	3.0192
Variance	17.4206	21.1505	21.7471	9.1158
Median	42.5038	43.6581	43.5677	29.4225
Geometric mean	43.0111	44.0132	43.9062	29.4225
95% HPD interval	[36.2412, 51.7211]	[36.1926, 53.2919]	[36.0603, 53.3643]	[20.714, 33.5511]
Auto-correlation time (ACT)	28259.7334	44580.6176	44558.5727	9309.9012
Effective sample size (ESS)	2388.6	1514.1	1514.9	7250.5
Number of samples	67501	67501	67501	67501

Stderr of mean = standard error of mean and Std dev = standard deviation.

Summary Statistic	(d) TMRCA estimates for N gene of TSWV-GA isolates			
	Peanut	Pepper	Tobacco	Tomato
Mean	26.5947	24.5814	26.595	25.9176
Stderr of mean	1.925	1.7875	1.9251	1.8767
Std dev	43.5427	40.5513	43.5512	42.5388
Variance	1895.9648	1644.4075	1896.7089	1809.5501
Median	15.0961	13.9812	15.0973	14.7111
Geometric mean	17.7157	16.309	17.7158	17.21
95% HPD interval	[3.9237, 80.0512]	[3.4663, 73.7259]	[3.9237, 80.0432]	[3.9237, 78.0173]

Auto-correlation time (ACT)	69418.3739	69018.7618	69401.0933	69135.2857
Effective sample size (ESS)	511.7	514.6	511.8	513.7
Number of samples	35518	35518	35518	35518

References:

Almási, A., et al. (2015). "Phylogenetic analysis of tomato spotted wilt virus (TSWV) NSs protein demonstrates the isolated emergence of resistance-breaking strains in pepper." Virus Genes **50**: 71-78.

Batuman, O., et al. (2017). "First report of a resistance-breaking strain of tomato spotted wilt virus infecting tomatoes with the Sw-5 tospovirus-resistance gene in California." Plant Disease **101**(4): 637-637.

Cho, S.-Y., et al. (2020). "First report of tomato spotted wilt virus infecting *Arachis hypogaea* in Korea." Journal of Plant Pathology **102**(1): 271-271.

De Haan, P., et al. (1989). "Molecular cloning and terminal sequence determination of the S and M RNAs of tomato spotted wilt virus." Journal of General Virology **70**(12): 3469-3473.

Debreczeni, D. E., et al. (2015). "Complete sequence of three different biotypes of tomato spotted wilt virus (wild type, tomato Sw-5 resistance-breaking and pepper Tsw resistance-breaking) from Spain." Archives of Virology **160**: 2117-2123.

Fidan, H. and N. Sari (2019). "Molecular characterization of resistance-breaking tomato spotted wilt virus (TSWV) isolate medium segment in tomato." Applied Ecology & Environmental Research **17**(2).

Gautam, S., et al. (2023). "First report of a resistance-breaking strain of tomato spotted wilt orthotospovirus infecting *Capsicum annuum* with the *Tsw* resistance gene in Texas." Plant Disease **107**(6).

Hu, Z.-Z., et al. (2011). "Complete genome sequence of a tomato spotted wilt virus isolate from China and comparison to other TSWV isolates of different geographic origin." Archives of Virology **156**: 1905-1908.

Jain, R., et al. (1998). "Molecular diagnosis of tomato spotted wilt tospovirus infection of peanut and other field and greenhouse crops." Plant Disease **82**(8): 900-904.

Kamran, A., et al. (2024). "Insights into the genetic variability and evolutionary dynamics of tomato spotted wilt orthotospovirus in China." BMC genomics **25**(1): 40.

Kwon, S.-J., et al. (2021). "Resistance-breaking tomato spotted wilt virus variant that recently occurred in pepper in South Korea is a genetic reassortant." Plant Disease **105**(10): 2771-2775.

Lahre, K., et al. (2023). "First report of resistance-breaking variants of tomato spotted wilt virus (TSWV) infecting tomatoes with the sw-5 resistance gene in north carolina." Plant Disease **107**(7): 2271.

Lee, J.-S., et al. (2011). "Complete genome sequences of three tomato spotted wilt virus isolates from tomato and pepper plants in Korea and their phylogenetic relationship to other TSWV isolates." Archives of Virology **156**: 725-728.

Lewandowski, D. J. and S. Adkins (2005). "The tubule-forming NSm protein from tomato spotted wilt virus complements cell-to-cell and long-distance movement of Tobacco mosaic virus hybrids." Virology **342**(1): 26-37.

Lopez, C., et al. (2011). "Evolutionary analysis of tomato Sw-5 resistance-breaking isolates of Tomato spotted wilt virus." Journal of General Virology **92**(1): 210-215.

Macedo, M. A., et al. (2024). "An all-out assault on a dominant resistance gene: Local emergence, establishment, and spread of strains of tomato spotted wilt orthotospovirus (TSWV) that overcome Sw-5b-mediated resistance in fresh market and processing tomatoes in California." Plos one **19**(7): e0305402.

Morca, A. F., et al. (2022). "Population analysis on tomato spotted wilt virus isolates inducing various symptoms on tomato, pepper, and *Chenopodium album* in Turkey." Physiological and Molecular Plant Pathology **118**: 101786.

Pappu, H., et al. (1998). "Sequence characteristics of natural populations of tomato spotted wilt tospovirus infecting flue-cured tobacco in Georgia." Virus Genes **17**: 169-177.

Pecman, A., et al. (2017). "Next generation sequencing for detection and discovery of plant viruses and viroids: comparison of two approaches." Frontiers in microbiology **8**: 1998.

Qiu, W., et al. (1998). "Tomato spotted wilttospovirusgenome reassortment and genome segment-specific adaptation." Virology **244**(1): 186-194.

Rodríguez-Negrete, E. A., et al. (2023). "A novel tomato spotted wilt virus isolate encoding a noncanonical NSm C118F substitution associated with *Sw-5* tomato gene resistance breaking." Molecular Plant Pathology **24**(10): 1300-1311.

Sundaraj, S., et al. (2014). "Host plant resistance against tomato spotted wilt virus in peanut (*Arachis hypogaea*) and its impact on susceptibility to the virus, virus population genetics, and vector feeding behavior and survival." Phytopathology **104**(2): 202-210.

Tentchev, D., et al. (2011). "Evolution and structure of tomato spotted wilt virus populations: evidence of extensive reassortment and insights into emergence processes." Journal of General Virology **92**(4): 961-973.

Terret-Welter, Z., et al. (2020). "Analysis of tomato spotted wilt virus RNA-dependent RNA polymerase adaptative evolution and constrained domains using homology protein structure modelling." Journal of General Virology **101**(3): 334-346.

Tsompana, M., et al. (2005). "The molecular population genetics of the tomato spotted wilt virus (TSWV) genome." Molecular Ecology **14**(1): 53-66.

Wylie, S. J., et al. (2015). "Differential responses to virus challenge of laboratory and wild accessions of Australian species of Nicotiana, and comparative analysis of RDR1 gene sequences." Plos one **10**(3): e0121787.

Zhang, Z., et al. (2016). "Identification of three new isolates of tomato spotted wilt virus from different hosts in China: molecular diversity, phylogenetic and recombination analyses." Virology journal **13**: 1-12.
